# Oxidative stress-mediated apoptosis via the *SLC23A2*-ascorbic acid interaction contributes to cleft lip development

**DOI:** 10.3389/fped.2025.1632778

**Published:** 2025-10-02

**Authors:** Bin Yin, Yi Chen Xu, Yong Lu

**Affiliations:** ^1^ Nanjing Stomatological Hospital Affiliated Hospital of Medical School, Institute of Stomatology, Nanjing University, Nanjing, China; ^2^ State Key Laboratory of Oral Diseases, West China Hospital of Stomatology, Sichuan University, Sichuan, China

**Keywords:** non-syndromic cleft lip only, association analysis, reactive oxygen species, gene–environment interaction, apoptosis

## Abstract

**Objectives:**

Non-syndromic cleft lip only (NSCLO) is a common subtype of cleft lip with/without cleft palate (CL/P). Previously, we found that *SLC23A2* is closely related to the occurrence of cleft palate through gene–environment interaction studies, but whether *SLC23A2* is related to the occurrence of cleft lip has not been reported.

**Design:**

First, the genotyping data of single-nucleotide polymorphisms (SNPs) at *SLC23A2* in 1,047 patients with NSCLO and 2,255 normal controls were extracted from two previous genome-wide association studies (GWASs) for an association analysis. Then, the interaction effect of *SLC23A2*, reactive oxygen species (ROS), and ascorbic acid (AA) on oxidative stress and apoptosis levels in the human oral epithelial-derived cell line (GMSM-K) and zebrafish was verified *in vitro* and *in vivo*. Finally, the mechanism of how *SLC23A2* is involved in the occurrence of cleft lip was initially explored using RNA sequencing.

**Results:**

The association analysis showed that 10 SNPs located at *SLC23A2* were significantly correlated with NSCLO. *In vitro* experiments have shown that knockdown of *SLC23A2* in GMSM-K inhibits the expression of *COL9A3* in the PI3K-Akt signaling pathway, promoting an increase in ROS and triggering increased apoptosis. The interaction results showed that the ROS and apoptosis levels increased in GMSM-K cells with normal *SLC23A2* gene function when stimulated by Sin-1 (exogenous ROS mimics) and ROS and apoptosis levels can be reduced by AA supplementation. GMSM-K cells became more sensitive to Sin-1, and AA supplementation was ineffective after *SLC23A2* knockdown. In addition, increased ROS and apoptosis levels were also observed in *slc23a2-MO* zebrafish, and could not be rescued by AA supplementation.

**Conclusion:**

*SLC23A2* was significantly associated with NSCLO. The *SLC23A2*/exogenous ROS/AA interaction is involved in lip and craniofacial development by influencing the levels of ROS and apoptosis.

## 1 Introduction

Non-syndromic cleft lip only (NSCLO) is a common subtype of cleft lip with/without cleft palate (CL/P). According to statistical data from 15,094,978 perinatal infants in China, the incidence of NSCLO is 0.56 per 1,000. Compared with cleft palate, the treatment of NSCLO still faces challenges, including a long treatment cycle, complex procedures, and high costs. Despite advances in surgical techniques, the fundamental repair methods have not significantly improved (1, 2). In addition, scarring and postoperative nasolabial deformities remain unavoidable, and patients often require multiple surgeries at different developmental stages to gradually correct facial morphology (1).

The etiology of NSCLO is complex, involving genetic factors, environmental factors, and their interactions, all of which play important roles in disease occurrence (3). In recent years, increasing attention has been paid to genetic research on NSCLO. Studies have reported that the rs642961 variant in the *IRF6* gene is associated with NSCLO in European (4) and Brazilian populations (5).

The rs12107 and rs2269529 variants in *MYH9* (6) and rs17563 and rs10130587 in *BMP4* (7) are linked to NSCLO in the Chinese Han population.

Current investigations into environmental factors primarily rely on epidemiological questionnaires. Factors such as smoking, alcohol consumption, hypoxia during pregnancy, and vitamin/folic acid supplementation have been implicated in cleft lip development (8, 9). Notably, Nakatomi et al. demonstrated that Msx1-deficient embryos develop a cleft lip following transient maternal hypoxia (10), providing direct evidence for gene–environment interactions in lip formation. However, few studies have explored gene–environment interactions in NSCLO. Our previous research screened genes associated with four environmental factors [smoking, alcohol consumption, hypoxia, and vitamin intake—including vitamins A, B9 (folic acid), C (ascorbic acid), D, and E] using genome-wide association study (GWAS) data. We identified that the vitamin C transporter gene *SLC23A2* is significantly linked to non-syndromic cleft palate only (NSCPO), with further experiments revealing its role in oxidative stress-mediated apoptosis. However, its potential influence on the occurrence of cleft lip remains unknown.

*SLC23A2* is critical for maintaining ascorbic acid (AA) levels in fetal and placental tissues. In *Slc23a2*^−/−^ mice, low AA levels resulted in fetal death, and increased oxidative stress and massive apoptosis were detected in the embryonic tissues of *Slc23a2*^−/−^ mice that survived the gestation period (11). SLC23A2 is a novel receptor-like transporter of AA, exhibiting dual functions: mediating AA uptake and activating the Janus kinase 2 (JAK2)/signal transducer and activator of transcription 2 (STAT2) signaling pathway. JAK2 activation synergistically promotes AA regulation in reactive oxygen species (ROS) scavenging (12). This suggests that the *SLC23A2* gene can influence oxidative stress by modulating cellular AA levels. Sustained oxidative stress may impair craniofacial development by increasing neural crest cell apoptosis (13). In addition, several studies have demonstrated that dysregulated redox homeostasis due to abnormal SLC23A2 function can disrupt various cellular biological processes. Downregulation of *SLC23A2* reduced bone marrow stromal cell (BMSC) attachment and spreading, whereas AA supplementation significantly rescued BMSCs from oxidative stress and enhanced wound closure (14). Impaired SLC23A2 activity leads to decreased AA uptake and ROS elimination, thereby affecting myoblast differentiation (15). *SLC23A2* also plays a crucial role in postnatal neuronal differentiation and neurite formation. Hippocampal neurons isolated from *Slc23a2*-knockout mice exhibited shorter neurites and reduced clustering of glutamate receptors (16).

**Figure 1:**
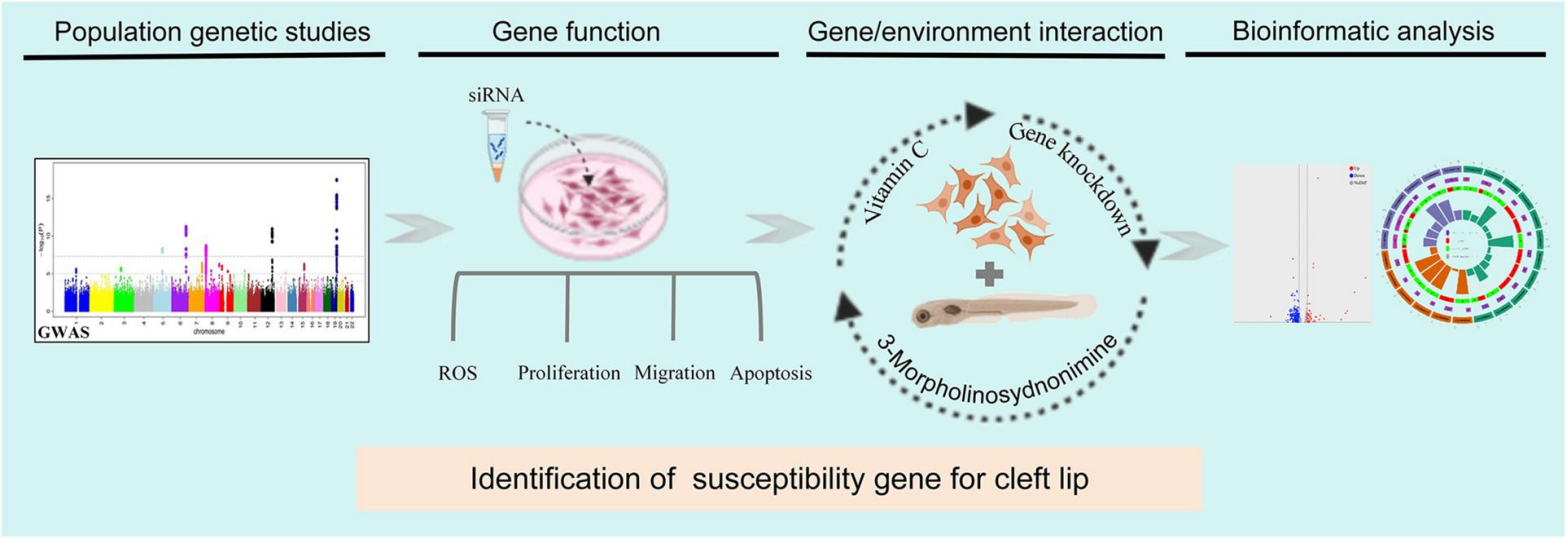
The interaction between *SLC23A2* and ascorbic acid plays a role in the occurrence of cleft lip by altering oxidative stress-mediated apoptosis. Flowchart of this study.

In the present study, we aimed to validate *SLC23A2* gene expression and construct an *in vitro* knockdown model to observe the phenotypic effects, preliminarily exploring its interaction with environmental factors in the development of NSCLO (Figure 1).

## 2 Materials and methods

### 2.1 Subject characterization and ethics statement

In this study, the genotyping data of single-nucleotide polymorphisms (SNPs) at *SLC23A2* in 1,047 patients with NSCLO and 2,255 normal controls were used from two previous GWASs (17, 18). The human subject study protocols were reviewed and approved by the institutional review board (IRB) of West China Hospital of Stomatology, Sichuan University, in 2016 (WCHSIRB-D-2016-012R1) and conformed to the Strengthening the Reporting of Observational Studies in Epidemiology (STROBE) guidelines. Written informed consent was obtained from recruited individuals of consenting age and from parents on behalf of their participating children.

### 2.2 Cell culture and transient knockdown

Considering the important role of oral epithelium in facial morphogenesis and its known association with NSCL/P, a human oral epithelial-derived cell line (GMSM-K, kindly gifted by Dr. Zhang from Peking University) was selected for functional analysis in our study (19). GMSM-K was cultured in Dulbecco's modified Eagle's medium (DMEM) supplemented with 10% fetal bovine serum (FBS; Gibco, United States) and 1% penicillin-streptomycin solution (Gibco, United States). Small interfering RNA (siRNA) targeting SLC23A2 (NM_005116.6) and negative control siRNA were both designed and synthesized by GenePharma (Shanghai, China). Information related to the siRNA can be found in the Supplementary Materials. Following the manufacturer's instructions, GMSM-K cells were seeded in a 6-well culture dish at a density of 1 × 10^5^ per well. When the cells reached a confluence of 70%–90%, siRNA was transfected into the GMSM-K cells using Lipofectamine 3000 (Thermo Scientific, United States). After 6 h, we replaced the Lipofectamine 3000-containing media with fresh complete media for further culturing. Following 48 or 72 h of transfection, the cells were collected to perform further examinations. The effective duration of siRNA-mediated gene silencing is 5–7 days.

### 2.3 Cell immunofluorescence

GMSM-K cells were seeded on a 6-well plate, rinsed with phosphate-buffered saline (PBS), and fixed with ice-cold methyl alcohol for 5 min. Next, the cells were permeabilized with 0.25% Triton X-100 for 5 min, washed with PBS twice, and blocked with 2.5% bovine serum albumin in PBS for 1 h. Antibodies against SLC23A2 (Novus, NBP2-13319) were diluted 150-fold with PBS and incubated at 4°C overnight.

### 2.4 ROS, superoxide dismutase activity, and glutathione/glutathione disulfide detection in cells

Cytosolic ROS were detected by staining the GMSM-K cells with 10 μM 2’,7’-dichlorodihydrofluorescein diacetate (DCFH-DA) (Sigama,Germany) in serum-free medium for 30 min at 37℃. The cells were washed twice with PBS. Under a fluorescence microscope, a fluorescein isothiocyanate (FITC) filter was used to observe fluorescence. Superoxide dismutase (SOD) activity and glutathione/glutathione disulfide (GSH/GSSG) were detected via a specific kit (Beyotime Biotechnology, China) following the manufacturer's instructions.

### 2.5 Proliferation assay

GMSM-K cells were seeded into 96-well plates at a density of 2 × 10^4^ cells/100 μL. At 21, 45, and 69 h after transfection, a mixture of 10 μL of Cell Counting Kit-8 (CCK-8) (APExBIO, United States) and 90μL DMEM was added to each well after removing the original medium and the cells were further incubated at 37℃ for 3 h. The optical density (OD) of the mixture was measured at a wavelength of 450 nm. The experiments were repeated three times and five parallel holes were set in each experiment.

### 2.6 Wound healing assay

GMSM-K cells were seeded into 6-well plates at a density of 2 × 10^5^ cells/2 mL. After transfection, five horizontal lines were scored on the bottom of the plates and the cells were scratched perpendicularly to the horizontal line using a sterile 20-µL pipette. The cells were then washed three times with PBS, placed under a microscope, and the medium was changed to 0.1% FBS experimental medium. The scratches were observed after incubation at 37°C for 0, 24, and 48 h.

### 2.7 Apoptosis assay

The apoptosis rate was evaluated using the Annexin V-PE/7-amino-actinomycin D (7-AAD) Apoptosis Detection Kit (Vazyme, China) according to the instructions from the manufacturer. The cells were seeded into 6-well tissue culture plates (2 × 10^5^ cells/well). Following treatment, the cells were collected, washed with PBS, and resuspended in 500 μL binding buffer. Then, 5 μL Annexin V-PE and 5 μL Annexin V-PE were added to the buffer and incubated at room temperature for 10 min in the dark. Cells were analyzed using flow cytometry (Thermo Fisher, United States) within 1 h. Flow Cytometry Standard (FCS) files were downloaded and analyzed using FlowJo software (version 10.4).

### 2.8 RNA sequencing, differential expression analysis, and Gene Ontology analysis

GMSM-K cells were transfected with a siRNA-negative control or siRNAs-SLC23A2 for 48 h. Then, RNA was extracted from the cells and RNA sequencing (RNA-seq) was performed using the BGISEQ-500 platform (BGI, China). Three biological replicates were included within each group. Differential gene expression analysis was performed using the DESeq2 method (|log2| ≥ 0.8, *q*-value ≤ 0.05), and Gene Ontology (GO)/Kyoto Encyclopedia of Genes and Genomes (KEGG) enrichment analysis was performed using ChiPlot (https://www.chiplot.online/).

### 2.9 RNA extraction, cDNA synthesis, and quantitative real-time PCR analysis

RNA was extracted 48 h after transfection using RNA-easyTM Isolation Reagent (Vazyme, China), and was then reverse-transcribed to cDNA using a PrimeScript™ RT reagent Kit (Takara Biotechnology, China). Real-time quantitative PCR (RT-qPCR) was performed using TB Green® Premix Ex Taq™ (Takara Biotechnology, China) on a LightCycler 480 System (Roche, Switzerland). All the experiments were performed in triplicate, each with three technical replicates. The results were calculated using the 2^−ΔΔCt^ equation, normalizing values to *GAPDH* within each sample. The primers used are shown in Supplementary Table 1.

### 2.10 Effects of the *SLC23A2*/exogenous ROS/AA interaction on cellular oxidative stress and apoptosis levels

A gene-environment interaction model in the GMSM-K cell line was established by knocking down *SLC23A2* and adding Sin-1 (20) and AA simultaneously. There were the following six groups: Negative control (NC), NC + Sin-1 (Aladdin, China), NC + Sin-1 + AA (Sigama, Germany), siRNA (si), si + Sin-1, and si + Sin-1 + AA. The detection methods of oxidative stress and apoptosis are the same as above.

### 2.11 Effects of the *slc23a2*/exogenous ROS/AA interaction on oxidative stress and apoptosis levels in zebrafish

All the animal experiments performed were approved by the Animal Ethical and Welfare Committee of Nanjing University with ID IACUC-D2310004 (2023.10.8). First, we used the previously verified morpholino (MO) technology to construct the *slc23a2*-knockdown zebrafish model. MO targeting at *slc23a2* (slc23a2-MO) (5'-GCACTGAATATGAAAAGATTGTACT-3’) was designed and produced by Gene Tools (United States). According to the preliminary experiment, the final concentration of slc23a2-MO was 2 ng/μL. Injections were carried out at the single cell stage, and after 8 h, unfertilized eggs and dead eggs were removed and replaced with fresh medium. At 48 h postfertilization (pf), the zebrafish embryos were collected, incubated with different concentrations of 3-Morpholinosydnonimine(Sin-1)(exogenous ROS mimics) and AA, treated with a 20.5 μM DCFH-DA probe (a chemically reduced form of fluorescein used as an indicator for ROS) and 5 μg/mL acridine orange (AO; an indicator for apoptosis) and then incubated in the dark at 28.5°C for 1 h. The embryos were then drenched with water three times, anaesthetized with 0.02% tricaine, and photographed under a fluorescence microscope with FITC filters.

### 2.12 Statistical analysis

The chi-square test and 95% confidence interval (95% CI) for the odds ratios were used to compare the allele frequency between the cases and controls. Each SNP was assessed using the Hardy–Weinberg equilibrium (HWE) and the minor allele frequency (MAF) was calculated. Moreover, the difference in allelic and genotypic frequencies of each SNP between the cases and normal controls was calculated using PLINK software (21). Pairwise linkage disequilibrium (LD), which shows both D′ and *R*^2^, was computed for all the SNPs using the Haploview program (http://www.broad.mit.edu/haploview/haploview). The results are shown as mean ± SD. A statistical analysis of the *in vivo* and *in vitro* experiments was performed using an unpaired two-tailed *t*-test in GraphPad Prism 8 software.

## 3 Results

### 3.1 SNPs within *SLC23A2* were significantly associated with NSCLO

**Figure 2:**
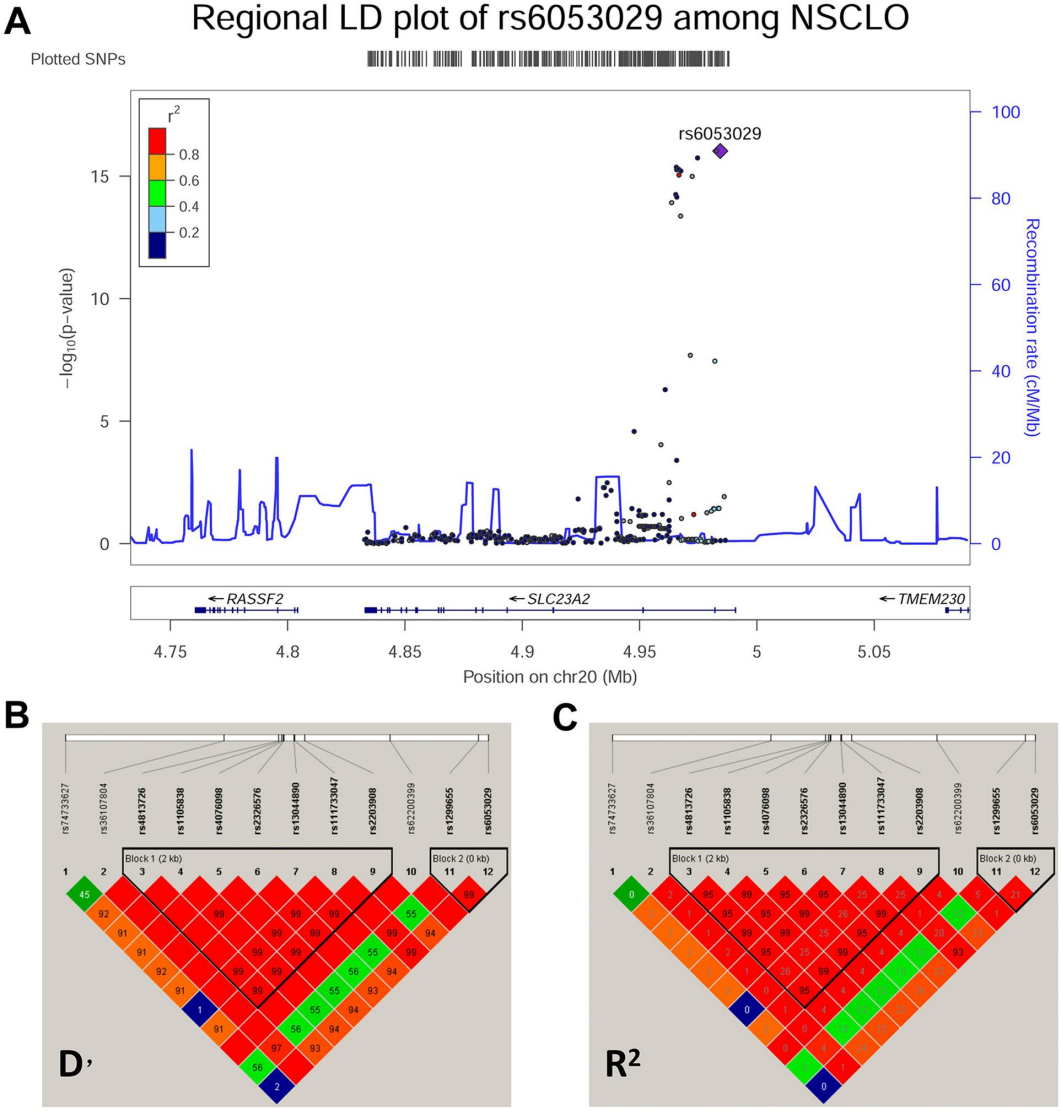
Association analysis and pairwise LD block of the SNPs at chromosome 20. **(A)** Association analysis and linkage disequilibrium in the Chr20 region. **(B,C)** Pairwise LD block of the SNPs at chromosome 20 in the cases and controls with NSCLO represented by D’ and *R*^2^, respectively.

A total of 306 common SNPs (MAF ≥0.01, with call rates >95%) that passed the HWE threshold (*p* > 0.05) were used in the association analysis (Supplementary Table 2). Both the allelic (Supplementary Table 3) and genotypic (Supplementary Table 4) association analyses indicated that 10 SNPs located at *SLC23A2* were significantly correlated with NSCLO, and were both adjusted for multiple corrections (*p* = 0.05/306). The pairwise LD results showed that rs6053029 was tightly linked to other SNPs and had the lowest *p*-value (9.44E-17) in NSCLO (Figure 2). Our previous RNA-seq results revealed that the expression of *SLC23A2* in the lip tissues was higher than that in the palate tissues (22) (Supplementary Figure 1).

### 3.2 *SLC23A2* knockdown altered ROS and cell biology in the GMSM-K cells

**Figure 3:**
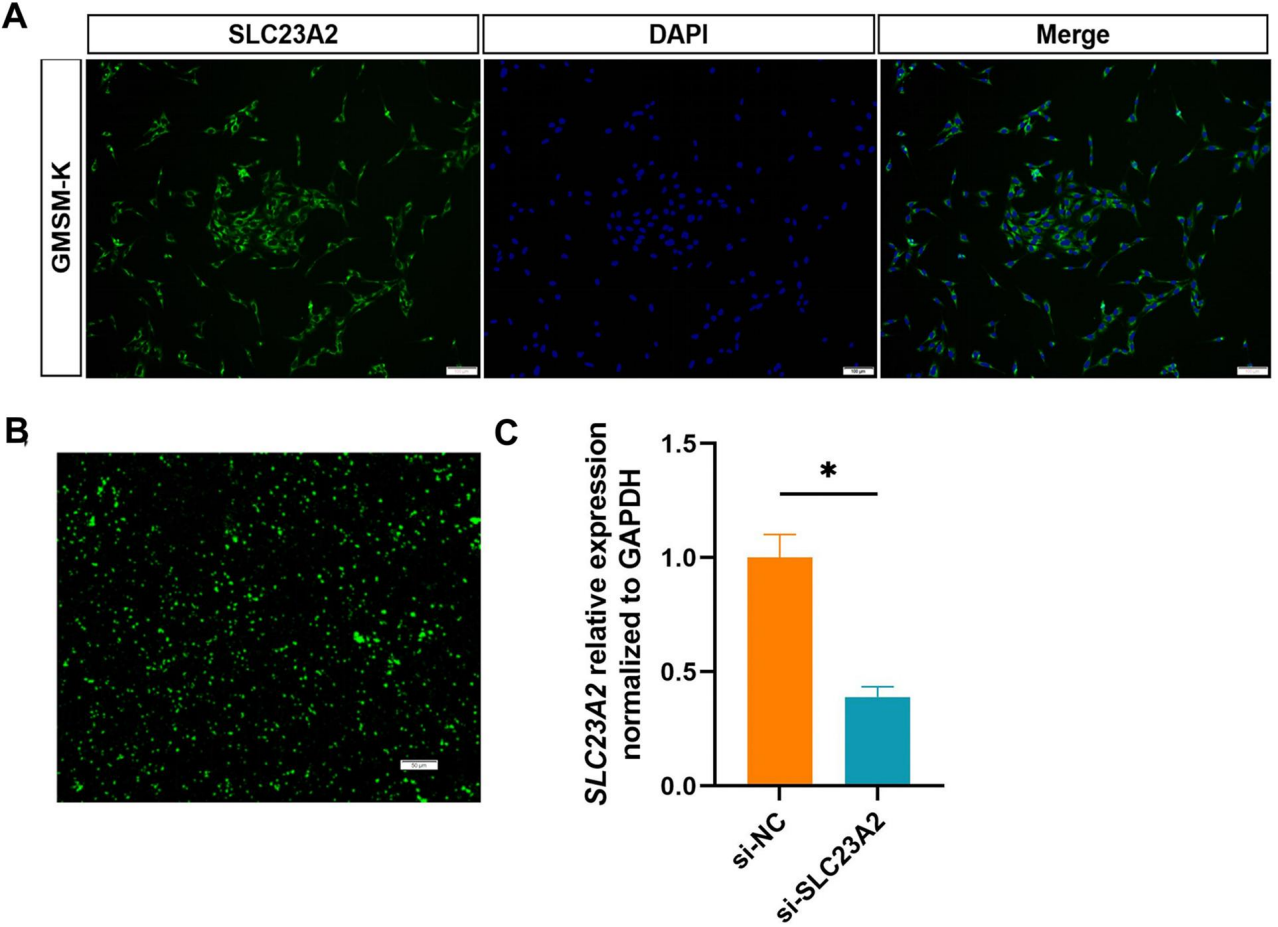
Construction of the *SLC23A2*-knockdown model in GMSM-K cells. **(A)** Immunofluorescence detection of the SLC23A2 protein in GMSM-K cells. **(B)** Transfected siRNA into GMSM-K cells. **(C)** Transfection efficiency was measured by qPCR. Error bars represent SD. *n* = 3; *, *P* < 0.05. Scale bar, 100 μm.

**Figure 4:**
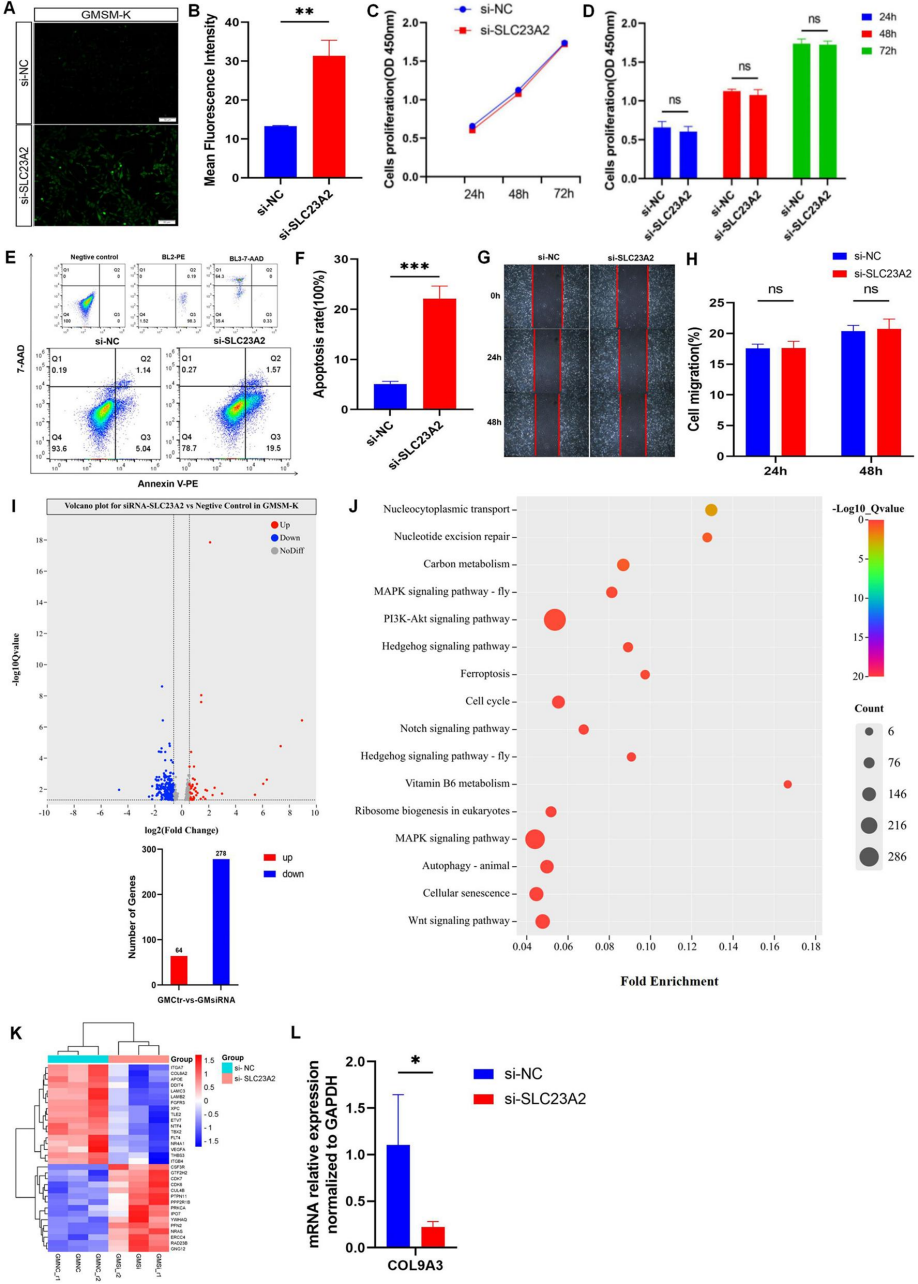
Changes in cell biology and ROS level in GMSM-K cells after *SLC23A2* knockdown. **(A,B)** ROS levels after knockdown of *SLC23A2*. **(C,D)** Cell proliferation after knockdown of *SLC23A2*. **(E,F)** Cell apoptosis after knockdown of *SLC23A2*. **(G,H)** Change in cell migration after *SLC23A2* knockdown. **(I)** Volcano plot for differential gene expression. **(J)** KEGG enrichment analysis of DEGs. **(K)**. The heat map of all DEGs, with genes related to cell proliferation, cell apoptosis, and cell cycle regulation given particular attention. **(L)** RT-qPCR verification result for the DEGs. Error bars represent SD. *n* = 3; ns, *P* > 0.05; **, *P* < 0.01; ***, *P* < 0.001.

We first detected the expression of *SLC23A2* in GMSM-K cells and found that SLC23A2 was expressed in the cell membrane (Figure 3A). Then, siRNA was used to construct an *SLC23A2*-knockdown GMSM-K cell model. The siRNA sequence information is as follows: F:GAGCCAUCCUGUCUUUAGATT, R:UCUAAAGACAGGAUGGCUCTT. The qPCR results showed that si-SLC23A2 effectively reduced the transcription level of the *SLC23A2* gene (Figures 3B,C). Studies have shown that knockout of *Slc23a2* can increase the level of oxidative stress in the embryonic tissues of mice, so we first examined the effect of *SLC23A2* knockdown on ROS levels. The data showed that the intracellular ROS levels in the GMSM-K cells significantly increased after *SLC23A2* gene knockdown (Figures 4A,B).

The CCK-8 assay showed that there was no significant change in the proliferation level of the GMSM-K cells after knocking down the *SLC23A2* gene (Figures 4C,D). The flow cytometry results showed that the early apoptosis levels of the GMSM-K cells increased significantly when the *SLC23A2* gene was knocked down (Figures 4E,F). However, cell migration was not significantly affected (Figures 4G,H).

### 3.3 *SLC23A2* influences biological processes in the etiology of lip and craniofacial abnormalities

To investigate the potential role of *SLC23A2* in the etiology of NSCLO, we performed RNA sequencing of GMSM-K cells with or without *SLC23A2* knockdown. Three biological replicates were set up in each group. The differential gene expression analysis identified 342 differentially expressed genes (DEGs) in total, including 64 upregulated genes and 278 downregulated genes (Figure 4I). KEGG analysis showed that a series of biological processes were enriched, including the PI3K-Akt signaling pathway, regulation of the cell cycle, cell senescence, and the Wnt signaling pathway (Figure 4J).

In order to further clarify the relationship between the DEGs enriched in GMSM-K cells and NSOC, we extracted the genotype data of differential genes from two previously published GWASs and conducted an association analysis with each NSOC subtype. The results showed that *IGFBP2* (rs9341191), *ITGB4* (rs820392, rs820390, rs820389, rs820387, rs866581, rs820388, and rs1008177), *LAMC3* (rs3780275), *LFNG* (rs10261289 and rs375386359), *NRARP* (rs34679617), *COL9A3* (rs2294995), 18 SNPs at *TLE2,* and 23 SNPs at *FLT4* were statistically significant (Supplementary Table 5). The genes that were statistically significant in the association analysis were verified by real-time fluorescent quantitative PCR, and the results showed that the expression of *COL9A3* in the PI3K-Akt signaling pathway was statistically different between the control group and the knockdown group (Figure 4L).

### 3.4 Effects of the *SLC23A2*/exogenous ROS/AA interaction on cellular oxidative stress levels and cellular biology in GMSM-K cells

This is the first time Sin-1 has been used in GMSM-K cells as an ROS mimicry drug. Therefore, we set the gradient according to the concentration of other cells in other studies, and determined the concentration by taking ROS detection and cell survival rate into account. The results from the DCFH-DA fluorescent probe showed that the effect of Sin-1 on the ROS levels in GMSM-K cells was dose-dependent. When the Sin-1 concentration was 400 , 600 , 800 , or 1,000 μM, there was no significant change in cell survival rate. Therefore, the intermediate concentration of Sin-1 of 800 μM was selected for the following experiments (Supplementary Figure 2A).

The concentration of AA changes its antioxidant effect. Therefore, we added different concentrations of AA to cells stimulated with 800 μM of Sin-1 and observed the altered ROS levels. The data showed that the antioxidant effect of AA was dose-dependent before 250 μM, with no significant increase in antioxidant activity over 250 μM. Thus, an AA concentration of 250 μMwas selected (Supplementary Figure 2B).

**Figure 5:**
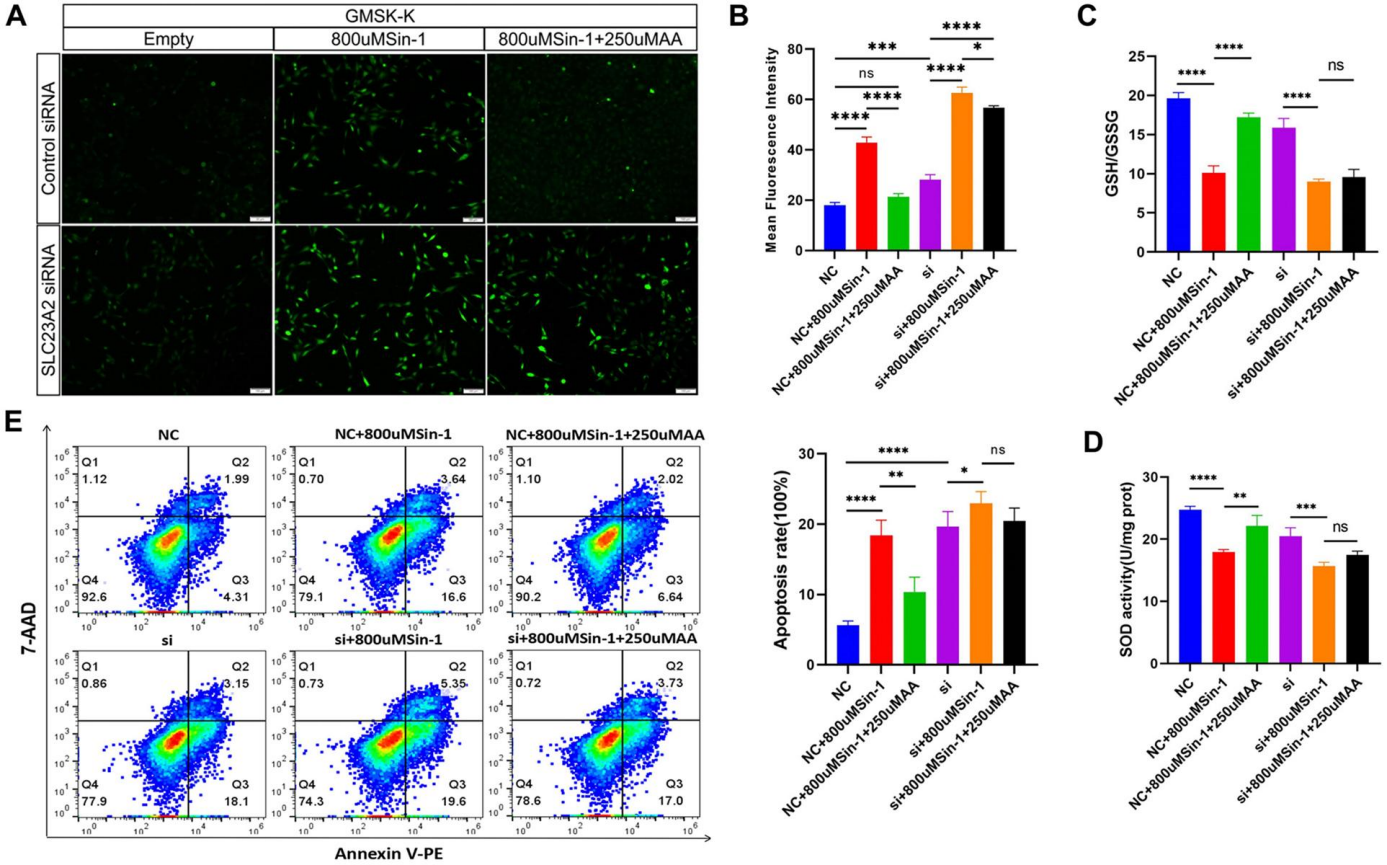
Effect of the *SLC23A2*/exogenous ROS/AA interaction on cellular oxidative stress level and cellular biology in GMSM-K cells. **(A,B)**. Effect of the *SLC23A2*/exogenous ROS/AA interaction on ROS levels. **(C)** Effect of the *SLC23A2*/exogenous ROS/AA interaction on GSH/GSSG. **(D)** Effect of the *SLC23A2*/exogenous ROS/AA interaction on SOD activity. **(E)** Effect of the *SLC23A2*/exogenous ROS/AA interaction on cell apoptosis. Error bars represent SD. *n* = 3; ns, *P* > 0.05; *, *P* < 0.05; **, *P* < 0.01; ***, *P* < 0.001; ****, *P* < 0.0001. Scale bar, 100 μm.

In order to explore the effect of AA antagonism on ROS levels in cells with normal and impaired *SLC23A2* gene function, we set up six groups for verification. The results showed that the normal *SLC23A2* gene function group (NC) had significantly increased ROS levels after Sin-1 stimulation (NC + 800 μM Sin-1). After incubation with AA (NC + 800 μM Sin-1 + 250 μM AA), the ROS level was lower than that after stimulation with Sin-1 alone, and there was no significant difference between the NC and NC + 800 μM Sin-1 + 250 μM AA groups. In the *SLC23A2* gene impaired group (si), the ROS level increased significantly after Sin-1 stimulation (si + 800 μM Sin-1), and the ROS level decreased slightly after coincubation with AA (si + 800 μMSin-1 + 250 μM AA) compared with that after pure Sin-1 stimulation (Figures 5A,B). The GSH/GSSG results showed that GSH/GSSG decreased significantly after Sin-1 stimulation in both the NC and si groups, while GSH/GSSG increased significantly after AA supplementation in the NC + 800 μM Sin-1 group and GSH/GSSG did not change significantly after AA supplementation in the si + 800 μM Sin-1 group (Figure 5C). The SOD activity detection results showed that the SOD activity of the NC and si groups significantly decreased after Sin-1 stimulation, while the SOD activity of the NC + 800 μM Sin-1 group significantly increased after AA supplementation and the SOD activity of the si + 800 μM Sin-1 group was not significantly changed after AA supplementation (Figure 5D).

After Sin-1 stimulation, apoptosis increased in both the NC and si groups. Apoptosis was significantly decreased after AA supplementation in the NC + 800 μM Sin-1 group, while there was no significant change in the si + 800 μM Sin-1 group (Figure 5E).

**Figure 6:**
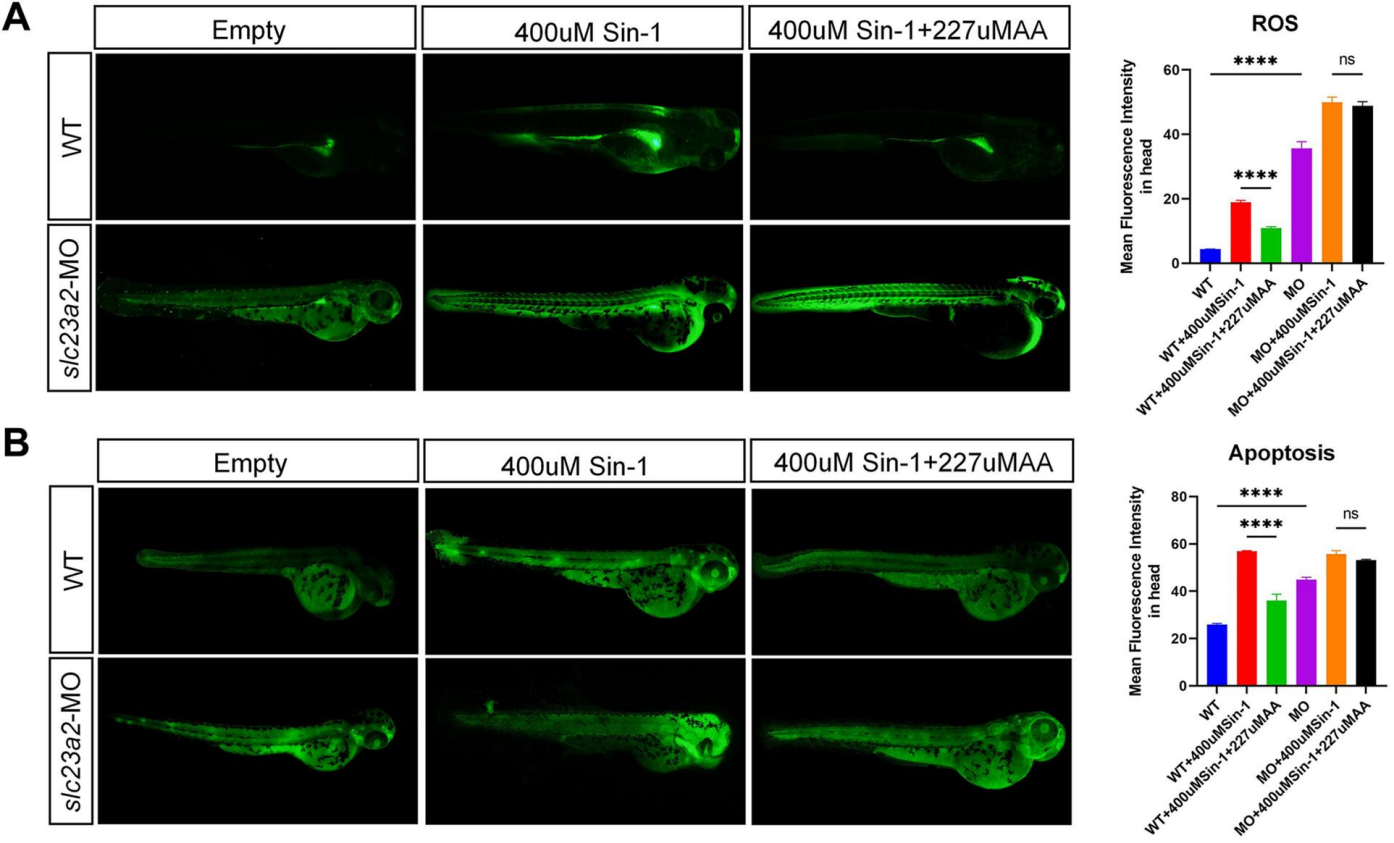
Effect of the *slc23a2*/exogenous ROS/AA interaction on oxidative stress and apoptosis in zebrafish cells. **(A)** Effect of the *slc23a2*/exogenous ROS/AA interaction on ROS levels. **(B)** Effect of the *slc23a2*/exogenous ROS/AA interaction on apoptosis. *slc23a2*-MO, *slc23a2*-knockdown zebrafish cells. Error bars represent SD. *n* = 3; ns, *P* > 0.05; ****, *P* < 0.0001.

To validate our findings *in vivo*, we detected the ROS and apoptosis levels in *slc23a2*-MO zebrafish via DCFH-DA and an AO fluorescent probe. Compared with the wild type (WT) group, the craniofacial ROS and apoptosis levels of the *slc23a2*-MO group were significantly increased. We consistently observed that 227 μM AA supplementation significantly decreased the ROS level and inhibited apoptosis induced by 400 μM Sin-1 stimulation in the WT group; however, there was no significant change in ROS and apoptosis levels after 227 μM AA supplementation in the *slc23a2*-MO group (Figures 6A,B).

## 4 Discussion

Gene–environment interactions as a mechanism for the occurrence of cleft lip and palate have been less extensively studied. In our previous study, we screened the correlation between four environmental factors, namely, maternal smoking, alcohol consumption, hypoxia, and vitamin intake [including vitamins A, B9 (folic acid), C (ascorbic acid), D, and E], and NSCPO. Among these, only the vitamin C transporter gene *SLC23A2* was significantly associated with NSCPO occurrence. In the present study, we further investigated the role of *SLC23A2* in NSCLO. The association analysis revealed that 10 SNPs within *SLC23A2* (rs36107804, rs4813726, rs1105838, rs4076098, rs2326576, rs13044890, rs111733047, rs2203908, rs62200399, and rs6053029) were significantly associated with NSCLO. Furthermore, our *in vitro* experiments demonstrated that GMSM-K cells exhibited elevated ROS levels and increased apoptosis upon *SLC23A2* knockdown, consistent with findings from mouse knockout models (11, 23).

Sustained oxidative stress can impair neural crest cell development through mechanisms such as DNA damage, p53 activation, and autophagy, ultimately contributing to craniofacial malformations (13). For example, Treacher Collins syndrome (TCS), a syndromic form of cleft lip and palate, is characterized by elevated oxidative stress *in vivo*. Tcof1 haploinsufficiency leads to oxidative stress-induced DNA damage and neuroepithelial cell death; however, maternal antioxidant treatment mitigates cell death and substantially prevents craniofacial anomalies (24). *SLC23A2* facilitates AA transport to protect tissues from oxidative damage. Furthermore, AA is essential for recycling other antioxidants, such as α-tocopherol (vitamin E) (25). Antioxidant supplementation (e.g., vitamin C or E) may reduce the incidence of developmental defects caused by excessive oxidative stress (26).

In this study, we used the triple interaction of *SLC23A2*/exogenous ROS/AA to simulate gene–environment interaction. Based on the literature review, we hypothesized that exogenous ROS stimulation in individuals with normal *SLC23A2* gene function would result in a mild disease phenotype, which could be alleviated by supplementation with the antioxidant AA, whereas exogenous ROS stimulation in individuals with abnormal *SLC23A2* function would further exacerbate the disease phenotype, and no significant improvement would be observed after supplementation with AA, as the efficiency of AA uptake would be reduced due to the dysfunction of *SLC23A2*. To test this hypothesis, we screened the optimal concentrations of Sin-1 and AA in GMSM-K cells, which were 800 and 250 μM, respectively. The interaction study revealed that cells with normal *SLC23A2* function were able to reduce the high ROS levels generated by Sin-1 stimulation after AA supplementation, while cells with abnormal *SLC23A2* function did not show any significant reduction in ROS levels after AA supplementation, which indicated that *SLC23A2* dysfunction led to the cells not being able to effectively utilize AA to counteract ROS. GSH/GSSG and SOD, the other two oxidative stress indexes, showed a consistent trend with ROS. We also detected apoptosis changes in the interaction study. The results showed that the apoptosis in both the wild-type cells and the *SLC23A2*-knockdown cells was increased by the Sin-1 stimulation and the degree of apoptosis in the *SLC23A2*-knockdown group was more significant. In addition, apoptosis in the wild-type cells was reduced by AA supplementation, whereas apoptosis in the cells in the *SLC23A2* knockdown group was not significantly improved by AA supplementation. Zebrafish are a common and useful scientific model organism for studying vertebrate development and gene function. Its genome has been completely sequenced. Compared with the human reference genome, approximately 70% of human genes have at least one obvious zebrafish homolog. Zebrafish currently provide a powerful animal model for studying craniomaxillofacial development (27). Increased ROS and apoptosis levels were also observed in the slc23a2-MO zebrafish cells and could not be rescued by AA supplementation. The results of the *in vitro* and *in vivo* experiments are consistent with our previous speculation that the triple interaction of *SLC23A2*/exogenous ROS/AA plays a role in lip and craniofacial development by modulating apoptotic alterations generated by oxidative stress. This part of the experiment will also provide a theoretical basis for pregnant mothers to supplement with antioxidants, such as AA, to prevent craniofacial deformities in their children. However, unfortunately, due to current technical limitations, we were unable to conduct further microscopic dissections and electron microscopy to observe the development of the lip. We plan to verify the craniofacial phenotype through subsequent experiments in mice.

To further explore the biological processes in which *SLC23A2* may be involved, we knocked down *SLC23A2* in GMSM-K cells and performed RNA-seq and GO and KEGG enrichment analyses, which showed that a number of DEGs were involved in a variety of biological processes, including the PI3K-Akt signaling pathway, regulation of the cell cycle, cell senescence, and the Wnt signaling pathway. In order to clarify the relationship between the above-mentioned DEGs enriched in GMSM-K cells and NSCLO, we extracted genotypic data of the DEGs from two previously published GWASs and performed an association analysis with various NSOC subtypes, and validated the genes that were statistically significantly different in the association analysis using RT-qPCR. The results showed that the expression of *COL9A3,* located in the PI3K-Akt signaling pathway, was statistically different between the control group and the knockdown group. The *COL9A3* gene is the pathogenic gene for Stickler syndrome with a cleft lip and palate phenotype (28).

In summary, this study revealed the role of oxidative stress-mediated apoptosis in the development of cleft lip through an association analysis, exploration of the signaling pathway mechanism involved in *SLC23A2*, and an analysis of the *SLC23A2*/ exogenous reactive oxygen species/AA interaction, providing a new theoretical basis for further improving the understanding of the etiology of NSCLO. Given this, our future research will focus on exploring whether it is necessary for pregnant women to supplement with antioxidants, such as vitamin C, in early pregnancy to reduce oxidative stress levels and prevent the occurrence of NSCLO. Furthermore, the interaction between autophagy, ROS, and apoptosis was not fully elucidated. Further research could utilize mouse knockout models to validate this mechanism.

## Data Availability

The datasets presented in this study can be found in online repositories. The names of the repository/repositories and accession number(s) can be found in the article/Supplementary Material.

## References

[1] Azouz V Ng M Patel N Murthy AS. Low incidence of maxillary hypoplasia in isolated cleft palate. Maxillofac Plast Reconstr Surg. (2020) 42(1):8. 10.1186/s40902-020-00252-9 32206667 PMC7083977

[2] Capelozza Filho L Normando AD da Silva Filho OG. Isolated influences of lip and palate surgery on facial growth: comparison of operated and unoperated male adults with UCLP. Cleft Palate Craniofac J. (1996) 33(1):51–6. 10.1597/1545-1569(1996)033<0051:IIOLAP>2.3.CO;2 8849859

[3] Nasreddine G El Hajj J Ghassibe-Sabbagh M. Orofacial clefts embryology, classification, epidemiology, and genetics. Mutat Res - Rev Mutat Res. (2021) 787:108373. 10.1016/j.mrrev.2021.108373 34083042

[4] Rahimov F Marazita ML Visel A Cooper ME Hitchler MJ Rubini M Disruption of an AP-2alpha binding site in an IRF6 enhancer is associated with cleft lip. Nat Genet. (2008) 40(11):1341–7. 10.1038/ng.242 18836445 PMC2691688

[5] Brito LA Bassi CF Masotti C Malcher C Rocha KM Schlesinger D IRF6 is a risk factor for nonsyndromic cleft lip in the Brazilian population. Am J Med Genet A. (2012) 158a(9):2170–5. 10.1002/ajmg.a.35526 22887868

[6] Wang Y Li D Xu Y Ma L Lu Y Wang Z Functional effects of SNPs in MYH9 and risks of nonsyndromic orofacial clefts. J Dent Res. (2018) 97(4):388–94. 10.1177/0022034517743930 29207917

[7] Hao J Gao R Wu W Hua L Chen Y Li F Association between BMP4 gene polymorphisms and cleft lip with or without cleft palate in a population from South China. Arch Oral Biol. (2018) 93:95–9. 10.1016/j.archoralbio.2018.05.015 29860186

[8] Jia ZL Shi B Chen CH Shi JY Wu J Xu X. Maternal malnutrition, environmental exposure during pregnancy and the risk of non-syndromic orofacial clefts. Oral Dis. (2011) 17(6):584–9. 10.1111/j.1601-0825.2011.01810.x 21535328

[9] Suazo J. Environmental factors in non-syndromic orofacial clefts: a review based on meta-analyses results. Oral Dis. (2022) 28(1):3–8. 10.1111/odi.13880 33872445

[10] Nakatomi M Ludwig KU Knapp M Kist R Lisgo S Ohshima H Msx1 deficiency interacts with hypoxia and induces a morphogenetic regulation during mouse lip development. Development. (2020) 147(21):dev189175. 10.1242/dev.189175 32467233

[11] Harrison FE Dawes SM Meredith ME Babaev VR Li L May JM. Low vitamin C and increased oxidative stress and cell death in mice that lack the sodium-dependent vitamin C transporter SVCT2. Free Radical Biol Med. (2010) 49(5):821–9. 10.1016/j.freeradbiomed.2010.06.008 20541602 PMC2916678

[12] Han Z Zhang Z Guan Y Chen B Yu M Zhang L New insights into vitamin C function: vitamin C induces JAK2 activation through its receptor-like transporter SVCT2. Int J Biol Macromol. (2021) 173:379–98. 10.1016/j.ijbiomac.2021.01.120 33484802

[13] Fitriasari S Trainor PA. Diabetes, oxidative stress, and DNA damage modulate cranial neural crest cell development and the phenotype variability of craniofacial disorders. Front Cell Dev Biol. (2021) 9:644410. 10.3389/fcell.2021.644410 34095113 PMC8174788

[14] Sangani R Pandya CD Bhattacharyya MH Periyasamy-Thandavan S Chutkan N Markand S Knockdown of SVCT2 impairs *in vitro* cell attachment, migration and wound healing in bone marrow stromal cells. Stem Cell Res. (2014) 12(2):354–63. 10.1016/j.scr.2013.11.002 24365600

[15] Fiorani M Scotti M Guidarelli A Burattini S Falcieri E Cantoni O. SVCT2-Dependent Plasma and mitochondrial membrane transport of ascorbic acid in differentiating myoblasts. Pharmacol Res. (2020) 159:105042. 10.1016/j.phrs.2020.105042 32580031

[16] Salazar K Espinoza F Cerda-Gallardo G Ferrada L Magdalena R Ramírez E SVCT2 Overexpression and ascorbic acid uptake increase cortical neuron differentiation, which is dependent on vitamin C recycling between neurons and astrocytes. Antioxidants (Basel). (2021) 10(9):1413. 10.3390/antiox10091413 34573045 PMC8465431

[17] Sun Y Huang Y Yin A Pan Y Wang Y Wang C Genome-wide association study identifies a new susceptibility locus for cleft lip with or without a cleft palate. Nat Commun. (2015) 6:6414. 10.1038/ncomms7414 25775280

[18] Huang L Jia Z Shi Y Du Q Shi J Wang Z Genetic factors define CPO and CLO subtypes of nonsyndromicorofacial cleft. PLoS Genet. (2019) 15(10):e1008357. 10.1371/journal.pgen.1008357 31609978 PMC6812857

[19] Liu H Duncan K Helverson A Kumari P Mumm C Xiao Y Analysis of zebrafish periderm enhancers facilitates identification of a regulatory variant near human KRT8/18. eLife. (2020) 9:e51325. 10.7554/eLife.51325 32031521 PMC7039683

[20] He Y Zhang Y Zhang D Zhang M Wang M Jiang Z 3-morpholinosydnonimine (SIN-1)-induced oxidative stress leads to necrosis in hypertrophic chondrocytes *in vitro*. Biomed Pharmacother. (2018) 106:1696–704. 10.1016/j.biopha.2018.07.128 30119244

[21] Purcell S Neale B Todd-Brown K Thomas L Ferreira MA Bender D PLINK: a tool set for whole-genome association and population-based linkage analyses. Am J Hum Genet. (2007) 81(3):559–75. 10.1086/519795 17701901 PMC1950838

[22] Ge B Lin Y Shi B Jia Z. Integrating transcriptomics and genomics to identify fibroblast growth factor/receptor candidate genes for non-syndromic orofacial cleft in Chinese. Arch Oral Biol. (2023) 153:105750. 10.1016/j.archoralbio.2023.105750 37348362

[23] Cao B Xia Y Cai Z Wang Z Tang C Song Y. Construction of a brain-specific SLC23A2 gene knockout mice model. Neuroscience. (2023) 524:137–48. 10.1016/j.neuroscience.2023.05.023 37330196

[24] Sakai D Dixon J Achilleos A Dixon M Trainor PA. Prevention of Treacher Collins syndrome craniofacial anomalies in mouse models via maternal antioxidant supplementation. Nat Commun. (2016) 7:10328. 10.1038/ncomms10328 26792133 PMC4735750

[25] Mock JT Chaudhari K Sidhu A Sumien N. The influence of vitamins E and C and exercise on brain aging. Exp Gerontol. (2017) 94:69–72. 10.1016/j.exger.2016.12.008 27939444 PMC5466517

[26] Hore TA von Meyenn F Ravichandran M Bachman M Ficz G Oxley D Retinol and ascorbate drive erasure of epigenetic memory and enhance reprogramming to naïve pluripotency by complementary mechanisms. Proc Natl Acad Sci U S A. (2016) 113(43):12202–7. 10.1073/pnas.1608679113 27729528 PMC5086989

[27] Li K Fan L Tian Y Lou S Li D Ma L Application of zebrafish in the study of craniomaxillofacial developmental anomalies. Birth Defects Res. (2022) 114(12):583–95. 10.1002/bdr2.2014 35437950

[28] Nixon TRW Richards AJ Martin H Alexander P Snead MP. Autosomal recessive Stickler syndrome. Genes (Basel). (2022) 13(7):1135. 10.3390/genes13071135 35885918 PMC9324312

