# Supplementary material for: Oxidative stress-mediated apoptosis via the SLC23A2-ascorbic acid interaction contributes to cleft lip development
Source: Front Pediatr. 2025 Oct 2;13:1632778. doi: 10.3389/fped.2025.1632778 (PMC12527864; doi:10.3389/fped.2025.1632778)
Supplement: Supplementary file 1 [file Table1.docx]

**Appendix Table 1** Primers for RT-qPCR

| Gene | Primer（5’-3’） |
| --- | --- |
| *SLC23A2*-F | AACACCACAGATGTTTCAGTTG |
| *SLC23A2*-R | GGACCGATGTACTTCAGTAGAG |
| *IGFBP2*-F | AACAGTGCAAGATGTCTCTGAACGG |
| *IGFBP2*-R | GCCTCCTGCTGCTCATTGTAGAAG |
| *ITGB4*-F | ATCGTGGAGCTGCTGGAGGAG |
| *ITGB4*-R | CGTCTTCTGGAACATCTTGGAGGTG |
| *TLE2*-F | TGGCGGTCGGAATGGAGAGTAG |
| *TLE2*-R | CAGGAGGCAAACTTCAGGGACAG |
| *NRARP*-F | TCGTGAAGCTGCTGGTCAAGTTC |
| *NRARP*-R | CTTCGCCTTGGTGATGAGATAGAGC |
| *FLT4*-F | CACGCAGAACTTGACCGACCTC |
| *FLT4*-R | TTCCTCCAGCAGCCTCTCGTC |
| *LFNG*-F | AGGTGACGCTGAGCTACGGTATG |
| *LFNG*-R | GGTACAGGTGGCAGTGGATGGAG |
| *LAMC3*-F | CACAGAGACACCGCCACCAAG |
| *LAMC3*-R | ACCCTTCCCTCCAGCAGATTCC |
| *GAPDH*-F | CTTTGGTATCGTGGAAGGACTC |
| *GAPDH*-R | GTAGAGGCAGGGATGATGTTCT |
