# Supplementary material for: Oxidative stress-mediated apoptosis via the SLC23A2-ascorbic acid interaction contributes to cleft lip development
Source: Front Pediatr. 2025 Oct 2;13:1632778. doi: 10.3389/fped.2025.1632778 (PMC12527864; doi:10.3389/fped.2025.1632778)
Supplement: Supplementary file 2 [file Table2.docx]

**Appendix Table 2** Hardy-Weinberg Equilibrium tests and MAF among normal controls of SNPs

| SNP | A1 | MAF | | HWE | | | |
| --- | --- | --- | --- | --- | --- | --- | --- |
|  |  | Control | NSCLO | GENO | O(HET) | E(HET) | P |
| rs1131382 | C | 0.4884 | 0.4887 | 424/894/475 | 0.4986 | 0.4996 | 0.9248 |
| rs35560557 | A | 0.1174 | 0.1277 | 30/372/1391 | 0.2075 | 0.2119 | 0.3723 |
| rs6052937 | A | 0.1112 | 0.1173 | 27/365/1401 | 0.2036 | 0.2064 | 0.5667 |
| rs79137959 | C | 0.0665 | 0.05796 | 6/217/1570 | 0.121 | 0.1196 | 0.8423 |
| rs6037991 | A | 0.08363 | 0.09142 | 13/295/1485 | 0.1645 | 0.163 | 0.8844 |
| rs16990309 | T | 0.09364 | 0.1004 | 20/314/1459 | 0.1751 | 0.1779 | 0.5059 |
| rs16990312 | G | 0.09564 | 0.1056 | 19/323/1451 | 0.1801 | 0.1811 | 0.7943 |
| rs16990314 | G | 0.08608 | 0.09614 | 18/290/1485 | 0.1617 | 0.1653 | 0.3892 |
| rs6037992 | C | 0.09564 | 0.1037 | 21/320/1452 | 0.1785 | 0.1815 | 0.4369 |
| rs3787455 | A | 0.2371 | 0.2521 | 107/659/1027 | 0.3675 | 0.3684 | 0.9489 |
| rs6037993 | C | 0.08608 | 0.09566 | 18/289/1486 | 0.1612 | 0.1648 | 0.3178 |
| rs6052940 | A | 0.09609 | 0.1051 | 20/322/1451 | 0.1796 | 0.1815 | 0.604 |
| rs6107541 | C | 0.09609 | 0.1051 | 20/322/1451 | 0.1796 | 0.1815 | 0.604 |
| rs6052942 | T | 0.09675 | 0.106 | 21/323/1449 | 0.1801 | 0.1828 | 0.518 |
| rs6037994 | T | 0.08563 | 0.09519 | 18/288/1487 | 0.1606 | 0.1644 | 0.3149 |
| rs3737321 | T | 0.2378 | 0.2484 | 109/660/1024 | 0.3681 | 0.3698 | 0.8481 |
| rs6052943 | A | 0.09431 | 0.1037 | 19/318/1456 | 0.1774 | 0.1788 | 0.6922 |
| rs6037998 | T | 0.0903 | 0.09566 | 21/303/1469 | 0.169 | 0.1739 | 0.2219 |
| rs6052944 | G | 0.0843 | 0.09378 | 17/285/1491 | 0.159 | 0.1621 | 0.3829 |
| rs11907979 | T | 0.08407 | 0.09378 | 17/284/1492 | 0.1584 | 0.1616 | 0.3803 |
| rs6038002 | C | 0.2378 | 0.2493 | 111/653/1029 | 0.3642 | 0.3689 | 0.6085 |
| rs12329577 | G | 0.1497 | 0.1508 | 39/467/1287 | 0.2605 | 0.2578 | 0.7144 |
| rs13045713 | G | 0.1793 | 0.1833 | 58/548/1187 | 0.3056 | 0.3018 | 0.6388 |
| rs6038004 | C | 0.2438 | 0.2502 | 114/674/1005 | 0.3759 | 0.3765 | 0.95 |
| rs146487465 | C | 0.04315 | 0.04995 | 2/162/1629 | 0.09035 | 0.0883 | 0.5861 |
| rs3787456 | A | 0.04782 | 0.03393 | 5/155/1633 | 0.08645 | 0.08779 | 0.4234 |
| rs1891649 | A | 0.2306 | 0.2333 | 99/662/1032 | 0.3692 | 0.3646 | 0.6502 |
| rs1110277 | G | 0.2407 | 0.2465 | 112/668/1013 | 0.3726 | 0.3737 | 0.8994 |
| rs8115867 | G | 0.2324 | 0.2337 | 102/664/1027 | 0.3703 | 0.3669 | 0.7476 |
| rs6038007 | T | 0.244 | 0.2418 | 112/683/998 | 0.3809 | 0.3779 | 0.8026 |
| rs8122118 | T | 0.2324 | 0.2337 | 102/664/1027 | 0.3703 | 0.3669 | 0.7476 |
| rs8123436 | T | 0.2318 | 0.2328 | 100/666/1027 | 0.3714 | 0.3664 | 0.606 |
| rs3787457 | T | 0.2318 | 0.2323 | 100/665/1028 | 0.3709 | 0.3661 | 0.6061 |
| rs3787458 | G | 0.2349 | 0.2356 | 107/662/1024 | 0.3692 | 0.3692 | 1 |
| rs3787459 | A | 0.2302 | 0.23 | 99/661/1033 | 0.3687 | 0.3643 | 0.6502 |
| rs78624304 | T | 0.06895 | 0.05749 | 9/220/1564 | 0.1227 | 0.1239 | 0.7009 |
| rs12481275 | A | 0.2404 | 0.2455 | 113/667/1013 | 0.372 | 0.374 | 0.8009 |
| rs6038010 | C | 0.2444 | 0.2413 | 114/680/999 | 0.3793 | 0.3782 | 0.9502 |
| rs2298174 | C | 0.2358 | 0.2304 | 100/671/1022 | 0.3742 | 0.3678 | 0.4805 |
| rs6116569 | C | 0.2647 | 0.2512 | 131/709/953 | 0.3954 | 0.3949 | 1 |
| rs4987219 | G | 0.2491 | 0.2385 | 121/676/996 | 0.377 | 0.3809 | 0.6644 |
| rs1715373 | G | 0.2602 | 0.2606 | 134/688/971 | 0.3837 | 0.391 | 0.4325 |
| rs79181767 | C | 0.02802 | 0.02026 | 1/103/1689 | 0.05745 | 0.05685 | 1 |
| rs6052956 | T | 0.2418 | 0.2375 | 109/674/1010 | 0.3759 | 0.3737 | 0.8497 |
| rs1776955 | C | 0.2754 | 0.2959 | 148/735/910 | 0.4099 | 0.4097 | 1 |
| rs75592697 | A | 0.02958 | 0.02875 | 0/118/1675 | 0.06581 | 0.06365 | 0.2599 |
| rs1629176 | A | 0.2491 | 0.2502 | 121/672/1000 | 0.3748 | 0.3798 | 0.5759 |
| rs1715374 | G | 0.25 | 0.2507 | 123/672/998 | 0.3748 | 0.3809 | 0.4954 |
| rs1776956 | G | 0.25 | 0.2526 | 123/671/999 | 0.3742 | 0.3807 | 0.4948 |
| rs1776957 | T | 0.2504 | 0.2531 | 124/671/998 | 0.3742 | 0.3812 | 0.4571 |
| rs1715375 | G | 0.25 | 0.2526 | 123/671/999 | 0.3742 | 0.3807 | 0.4948 |
| rs1776958 | G | 0.2616 | 0.2611 | 137/685/971 | 0.382 | 0.3918 | 0.3054 |
| rs1776959 | G | 0.2504 | 0.246 | 120/683/990 | 0.3809 | 0.3823 | 0.9017 |
| rs1776960 | T | 0.2504 | 0.246 | 119/684/990 | 0.3815 | 0.382 | 0.9507 |
| rs1715376 | C | 0.2527 | 0.2531 | 124/671/998 | 0.3742 | 0.3812 | 0.4571 |
| rs1776961 | C | 0.01179 | 0.01037 | 0/40/1753 | 0.02231 | 0.02206 | 1 |
| rs6052961 | T | 0.06717 | 0.06786 | 12/218/1563 | 0.1216 | 0.1259 | 0.184 |
| rs1776963 | T | 0.1957 | 0.1767 | 72/552/1169 | 0.3079 | 0.3128 | 0.4971 |
| rs1715377 | A | 0.1428 | 0.1263 | 30/436/1327 | 0.2432 | 0.2384 | 0.4293 |
| rs1715378 | A | 0.1357 | 0.123 | 27/421/1345 | 0.2348 | 0.2298 | 0.4112 |
| rs1715379 | G | 0.2758 | 0.2658 | 141/713/939 | 0.3977 | 0.401 | 0.724 |
| rs1776964 | G | 0.2742 | 0.2653 | 140/710/943 | 0.396 | 0.3997 | 0.6796 |
| rs1715380 | C | 0.1617 | 0.1437 | 41/486/1266 | 0.2711 | 0.2666 | 0.5353 |
| rs6052962 | A | 0.02269 | 0.01838 | 1/76/1716 | 0.04239 | 0.04256 | 0.5751 |
| rs1715381 | T | 0.2702 | 0.2554 | 142/692/959 | 0.3859 | 0.3962 | 0.2832 |
| rs1776965 | T | 0.1608 | 0.1404 | 40/484/1269 | 0.2699 | 0.2651 | 0.4767 |
| rs77395977 | T | 0.1323 | 0.1126 | 26/409/1358 | 0.2281 | 0.2241 | 0.5267 |
| rs1715382 | C | 0.1323 | 0.1126 | 26/409/1358 | 0.2281 | 0.2241 | 0.5267 |
| rs1715383 | G | 0.1648 | 0.1442 | 43/492/1258 | 0.2744 | 0.2704 | 0.6001 |
| rs1715384 | T | 0.2716 | 0.2573 | 142/697/954 | 0.3887 | 0.3975 | 0.3726 |
| rs2748896 | A | 0.1688 | 0.1503 | 48/496/1249 | 0.2766 | 0.2757 | 0.9319 |
| rs8125804 | T | 0.05249 | 0.05796 | 5/187/1601 | 0.1043 | 0.1038 | 1 |
| rs8125856 | T | 0.09186 | 0.09378 | 23/296/1474 | 0.1651 | 0.1726 | 0.07404 |
| rs4815754 | A | 0.1303 | 0.1107 | 26/402/1365 | 0.2242 | 0.2212 | 0.6688 |
| rs1776966 | A | 0.1246 | 0.1056 | 18/394/1381 | 0.2197 | 0.2111 | 0.09266 |
| rs1715385 | G | 0.2705 | 0.2535 | 143/688/962 | 0.3837 | 0.3957 | 0.2099 |
| rs4813723 | C | 0.276 | 0.2606 | 152/694/947 | 0.3871 | 0.4017 | 0.1263 |
| rs1715386 | T | 0.1281 | 0.1079 | 24/399/1370 | 0.2225 | 0.2182 | 0.4497 |
| rs74333140 | C | 0.02224 | 0.01791 | 1/75/1717 | 0.04183 | 0.04202 | 0.5656 |
| rs1715387 | T | 0.02113 | 0.01838 | 1/73/1719 | 0.04071 | 0.04095 | 0.5463 |
| rs1935972 | A | 0.2086 | 0.1998 | 89/571/1133 | 0.3185 | 0.3305 | 0.1331 |
| rs6084932 | C | 0.2084 | 0.1998 | 90/568/1135 | 0.3168 | 0.3302 | 0.08624 |
| rs77881678 | T | 0.02069 | 0.01791 | 1/71/1721 | 0.0396 | 0.03989 | 0.5267 |
| rs1776967 | T | 0.02157 | 0.01838 | 1/75/1717 | 0.04183 | 0.04202 | 0.5656 |
| rs1776968 | C | 0.2071 | 0.1979 | 88/567/1138 | 0.3162 | 0.3285 | 0.1139 |
| rs1614554 | C | 0.02135 | 0.01838 | 1/73/1719 | 0.04071 | 0.04095 | 0.5463 |
| rs1776970 | C | 0.02135 | 0.01838 | 1/73/1719 | 0.04071 | 0.04095 | 0.5463 |
| rs1715362 | T | 0.02135 | 0.01838 | 1/73/1719 | 0.04071 | 0.04095 | 0.5463 |
| rs1715364 | T | 0.2031 | 0.1956 | 84/557/1152 | 0.3107 | 0.3226 | 0.1239 |
| rs1715365 | C | 0.2077 | 0.1998 | 87/569/1137 | 0.3173 | 0.3285 | 0.1506 |
| rs1715366 | C | 0.2077 | 0.1998 | 88/569/1136 | 0.3173 | 0.3292 | 0.1318 |
| rs1715367 | C | 0.2073 | 0.1989 | 88/565/1140 | 0.3151 | 0.3279 | 0.09795 |
| rs2681107 | C | 0.2071 | 0.1989 | 88/565/1140 | 0.3151 | 0.3279 | 0.09795 |
| rs75072655 | A | 0.02825 | 0.04147 | 1/113/1679 | 0.06302 | 0.06208 | 1 |
| rs3787469 | C | 0.05316 | 0.04807 | 2/182/1609 | 0.1015 | 0.09836 | 0.2308 |
| rs1715368 | C | 0.02157 | 0.01885 | 1/75/1717 | 0.04183 | 0.04202 | 0.5656 |
| rs74912238 | A | 0.02157 | 0.01838 | 1/75/1717 | 0.04183 | 0.04202 | 0.5656 |
| rs73897144 | T | 0.02157 | 0.01838 | 1/75/1717 | 0.04183 | 0.04202 | 0.5656 |
| rs73894175 | G | 0.0536 | 0.04854 | 2/184/1607 | 0.1026 | 0.09936 | 0.2315 |
| rs117968930 | G | 0.0536 | 0.04807 | 2/184/1607 | 0.1026 | 0.09936 | 0.2315 |
| rs1776971 | C | 0.02202 | 0.02026 | 1/77/1715 | 0.04294 | 0.04309 | 0.5846 |
| rs80244584 | G | 0.05405 | 0.04854 | 2/186/1605 | 0.1037 | 0.1004 | 0.2333 |
| rs1776972 | C | 0.02202 | 0.02026 | 1/77/1715 | 0.04294 | 0.04309 | 0.5846 |
| rs76415638 | T | 0.0536 | 0.04807 | 2/184/1607 | 0.1026 | 0.09936 | 0.2315 |
| rs1776973 | C | 0.02202 | 0.02026 | 1/77/1715 | 0.04294 | 0.04309 | 0.5846 |
| rs939257 | C | 0.02224 | 0.02026 | 1/77/1715 | 0.04294 | 0.04309 | 0.5846 |
| rs939258 | A | 0.07585 | 0.0688 | 8/251/1534 | 0.14 | 0.1378 | 0.6089 |
| rs939259 | A | 0.02157 | 0.01932 | 1/75/1717 | 0.04183 | 0.04202 | 0.5656 |
| rs1715370 | T | 0.02157 | 0.01932 | 1/75/1717 | 0.04183 | 0.04202 | 0.5656 |
| rs76476203 | G | 0.02113 | 0.01885 | 1/73/1719 | 0.04071 | 0.04095 | 0.5463 |
| rs1776977 | T | 0.0218 | 0.01932 | 1/75/1717 | 0.04183 | 0.04202 | 0.5656 |
| rs116915305 | A | 0.0536 | 0.04854 | 2/184/1607 | 0.1026 | 0.09936 | 0.2315 |
| rs1715372 | G | 0.149 | 0.1466 | 45/444/1304 | 0.2476 | 0.2535 | 0.3508 |
| rs138782359 | T | 0.0754 | 0.06786 | 8/249/1536 | 0.1389 | 0.1369 | 0.7286 |
| rs56671692 | A | 0.0536 | 0.04854 | 2/184/1607 | 0.1026 | 0.09936 | 0.2315 |
| rs59971708 | A | 0.0536 | 0.04854 | 2/184/1607 | 0.1026 | 0.09936 | 0.2315 |
| rs1776978 | A | 0.0218 | 0.01932 | 1/75/1717 | 0.04183 | 0.04202 | 0.5656 |
| rs73893863 | A | 0.05427 | 0.04854 | 3/185/1605 | 0.1032 | 0.1009 | 0.48 |
| rs1628664 | A | 0.02157 | 0.01932 | 1/75/1717 | 0.04183 | 0.04202 | 0.5656 |
| rs3761240 | A | 0.0536 | 0.04854 | 2/184/1607 | 0.1026 | 0.09936 | 0.2315 |
| rs79530091 | C | 0.02091 | 0.01885 | 1/72/1720 | 0.04016 | 0.04042 | 0.5365 |
| rs2946682 | G | 0.07585 | 0.06833 | 8/251/1534 | 0.14 | 0.1378 | 0.6089 |
| rs55793575 | A | 0.0536 | 0.04854 | 2/184/1607 | 0.1026 | 0.09936 | 0.2315 |
| rs189470744 | T | 0.0536 | 0.04807 | 2/184/1607 | 0.1026 | 0.09936 | 0.2315 |
| rs181793493 | A | 0.02157 | 0.01979 | 1/75/1717 | 0.04183 | 0.04202 | 0.5656 |
| rs1715360 | C | 0.0218 | 0.01979 | 1/75/1717 | 0.04183 | 0.04202 | 0.5656 |
| rs1343095 | G | 0.07585 | 0.06786 | 8/251/1534 | 0.14 | 0.1378 | 0.6089 |
| rs6052972 | A | 0.04004 | 0.04383 | 5/141/1647 | 0.07864 | 0.08067 | 0.2424 |
| rs13037855 | T | 0.0278 | 0.02262 | 1/92/1700 | 0.05131 | 0.05105 | 1 |
| rs113257858 | T | 0.06561 | 0.05985 | 4/229/1560 | 0.1277 | 0.1234 | 0.1794 |
| rs111806610 | A | 0.01535 | 0.008954 | 1/59/1733 | 0.03291 | 0.03344 | 0.4049 |
| rs1715392 | T | 0.06628 | 0.05985 | 4/232/1557 | 0.1294 | 0.1249 | 0.1813 |
| rs1776947 | G | 0.06606 | 0.05985 | 4/231/1558 | 0.1288 | 0.1244 | 0.1804 |
| rs1519862 | A | 0.06628 | 0.05985 | 4/232/1557 | 0.1294 | 0.1249 | 0.1813 |
| rs3914810 | C | 0.258 | 0.2436 | 135/667/991 | 0.372 | 0.386 | 0.1262 |
| rs1519864 | G | 0.06584 | 0.05985 | 4/230/1559 | 0.1283 | 0.1239 | 0.1797 |
| rs12625783 | C | 0.01646 | 0.01461 | 0/61/1732 | 0.03402 | 0.03344 | 1 |
| rs1776948 | G | 0.1933 | 0.1814 | 75/549/1169 | 0.3062 | 0.3139 | 0.2928 |
| rs1519865 | A | 0.06628 | 0.05985 | 4/232/1557 | 0.1294 | 0.1249 | 0.1813 |
| rs1715395 | G | 0.06628 | 0.05985 | 4/232/1557 | 0.1294 | 0.1249 | 0.1813 |
| rs1776950 | A | 0.06628 | 0.05985 | 4/232/1557 | 0.1294 | 0.1249 | 0.1813 |
| rs1715397 | G | 0.0665 | 0.05985 | 4/233/1556 | 0.1299 | 0.1254 | 0.1825 |
| rs1776952 | T | 0.0665 | 0.05985 | 4/233/1556 | 0.1299 | 0.1254 | 0.1825 |
| rs1715361 | A | 0.0665 | 0.05985 | 4/233/1556 | 0.1299 | 0.1254 | 0.1825 |
| rs7260796 | C | 0.01557 | 0.008954 | 1/61/1731 | 0.03402 | 0.03452 | 0.4255 |
| rs57215863 | T | 0.01557 | 0.008954 | 1/61/1731 | 0.03402 | 0.03452 | 0.4255 |
| rs16990455 | T | 0.01557 | 0.008954 | 1/61/1731 | 0.03402 | 0.03452 | 0.4255 |
| rs79494287 | A | 0.0149 | 0.008011 | 1/58/1734 | 0.03235 | 0.0329 | 0.3946 |
| rs113678532 | A | 0.01579 | 0.008483 | 1/62/1730 | 0.03458 | 0.03506 | 0.4358 |
| rs2681109 | A | 0.04226 | 0.03817 | 0/149/1644 | 0.0831 | 0.07965 | 0.0714 |
| rs75511237 | C | 0.0149 | 0.008011 | 1/59/1733 | 0.03291 | 0.03344 | 0.4049 |
| rs1776953 | T | 0.02825 | 0.01838 | 0/110/1683 | 0.06135 | 0.05947 | 0.4108 |
| rs190393511 | C | 0.02024 | 0.01084 | 0/77/1716 | 0.04294 | 0.04202 | 1 |
| rs6107559 | G | 0.361 | 0.4006 | 232/829/732 | 0.4624 | 0.4611 | 0.9591 |
| rs2681110 | T | 0.3957 | 0.426 | 285/856/652 | 0.4774 | 0.4791 | 0.8825 |
| rs6084944 | A | 0.3694 | 0.3869 | 244/844/705 | 0.4707 | 0.4669 | 0.7616 |
| rs6139587 | T | 0.3679 | 0.3836 | 240/845/708 | 0.4713 | 0.4659 | 0.6484 |
| rs6052988 | C | 0.3679 | 0.385 | 240/845/708 | 0.4713 | 0.4659 | 0.6484 |
| rs6038020 | T | 0.367 | 0.3812 | 240/844/709 | 0.4707 | 0.4658 | 0.6851 |
| rs6052990 | C | 0.367 | 0.3817 | 240/844/709 | 0.4707 | 0.4658 | 0.6851 |
| rs6038021 | A | 0.367 | 0.3817 | 240/844/709 | 0.4707 | 0.4658 | 0.6851 |
| rs6052991 | G | 0.367 | 0.3817 | 240/844/709 | 0.4707 | 0.4658 | 0.6851 |
| rs6052992 | C | 0.367 | 0.3817 | 240/844/709 | 0.4707 | 0.4658 | 0.6851 |
| rs6139588 | A | 0.367 | 0.3817 | 240/844/709 | 0.4707 | 0.4658 | 0.6851 |
| rs6052993 | G | 0.3692 | 0.3817 | 246/841/706 | 0.469 | 0.4671 | 0.8795 |
| rs6052994 | T | 0.3692 | 0.3817 | 246/841/706 | 0.469 | 0.4671 | 0.8795 |
| rs6052995 | C | 0.3692 | 0.3817 | 246/841/706 | 0.469 | 0.4671 | 0.8795 |
| rs6052996 | G | 0.369 | 0.3817 | 246/840/707 | 0.4685 | 0.4669 | 0.9195 |
| rs6038022 | T | 0.367 | 0.3812 | 240/844/709 | 0.4707 | 0.4658 | 0.6851 |
| rs6038023 | A | 0.367 | 0.3812 | 240/844/709 | 0.4707 | 0.4658 | 0.6851 |
| rs2748902 | G | 0.3688 | 0.3883 | 246/840/707 | 0.4685 | 0.4669 | 0.9195 |
| rs2681113 | C | 0.3688 | 0.3888 | 246/840/707 | 0.4685 | 0.4669 | 0.9195 |
| rs6052998 | A | 0.367 | 0.3817 | 240/844/709 | 0.4707 | 0.4658 | 0.6851 |
| rs2748901 | A | 0.3783 | 0.4204 | 256/855/682 | 0.4769 | 0.4718 | 0.6888 |
| rs1879176 | C | 0.367 | 0.3817 | 240/844/709 | 0.4707 | 0.4658 | 0.6851 |
| rs1879177 | T | 0.3668 | 0.3817 | 240/843/710 | 0.4702 | 0.4656 | 0.7226 |
| rs6038025 | T | 0.3665 | 0.3812 | 239/844/710 | 0.4707 | 0.4655 | 0.6483 |
| rs1519866 | G | 0.3661 | 0.3803 | 239/845/709 | 0.4713 | 0.4656 | 0.648 |
| rs13037458 | C | 0.4237 | 0.4599 | 341/864/588 | 0.4819 | 0.4905 | 0.4702 |
| rs35731094 | A | 0.05027 | 0.041 | 4/187/1602 | 0.1043 | 0.1028 | 0.8165 |
| rs2681116 | C | 0.4259 | 0.4665 | 345/865/583 | 0.4824 | 0.4912 | 0.4707 |
| rs6139591 | A | 0.3643 | 0.3779 | 242/836/715 | 0.4663 | 0.4652 | 0.9595 |
| rs114776261 | T | 0.05027 | 0.041 | 4/187/1602 | 0.1043 | 0.1028 | 0.8165 |
| rs6053002 | C | 0.3643 | 0.3779 | 242/836/715 | 0.4663 | 0.4652 | 0.9595 |
| rs187777103 | T | 0.05027 | 0.041 | 4/187/1602 | 0.1043 | 0.1028 | 0.8165 |
| rs201341095 | C | 0.05027 | 0.041 | 4/187/1602 | 0.1043 | 0.1028 | 0.8165 |
| rs115233603 | T | 0.05027 | 0.041 | 4/187/1602 | 0.1043 | 0.1028 | 0.8165 |
| rs7263163 | T | 0.4239 | 0.4599 | 342/863/588 | 0.4813 | 0.4906 | 0.4411 |
| rs76480995 | T | 0.05027 | 0.041 | 4/187/1602 | 0.1043 | 0.1028 | 0.8165 |
| rs6139592 | A | 0.4226 | 0.4599 | 339/863/591 | 0.4813 | 0.4901 | 0.4412 |
| rs79774064 | C | 0.05004 | 0.041 | 4/186/1603 | 0.1037 | 0.1023 | 0.816 |
| rs6139593 | T | 0.423 | 0.4599 | 340/863/590 | 0.4813 | 0.4903 | 0.4411 |
| rs12480138 | G | 0.3641 | 0.3784 | 241/835/717 | 0.4657 | 0.4648 | 0.9595 |
| rs12481301 | T | 0.3641 | 0.3784 | 241/835/717 | 0.4657 | 0.4648 | 0.9595 |
| rs192759748 | T | 0.05004 | 0.041 | 4/186/1603 | 0.1037 | 0.1023 | 0.816 |
| rs58061452 | T | 0.3645 | 0.3779 | 243/833/717 | 0.4646 | 0.4651 | 0.9595 |
| rs6053003 | A | 0.423 | 0.4599 | 340/863/590 | 0.4813 | 0.4903 | 0.4411 |
| rs146463412 | C | 0.05004 | 0.041 | 4/186/1603 | 0.1037 | 0.1023 | 0.816 |
| rs577998756 | T | 0.04982 | 0.04053 | 4/185/1604 | 0.1032 | 0.1018 | 0.8155 |
| rs545078767 | C | 0.04982 | 0.04053 | 4/185/1604 | 0.1032 | 0.1018 | 0.8155 |
| rs553897675 | T | 0.04938 | 0.04053 | 4/183/1606 | 0.1021 | 0.1009 | 0.8149 |
| rs572298297 | A | 0.04938 | 0.04053 | 4/183/1606 | 0.1021 | 0.1009 | 0.8149 |
| rs542764191 | T | 0.05004 | 0.041 | 4/186/1603 | 0.1037 | 0.1023 | 0.816 |
| rs192725418 | A | 0.04938 | 0.041 | 4/183/1606 | 0.1021 | 0.1009 | 0.8149 |
| rs531873467 | T | 0.04938 | 0.041 | 4/183/1606 | 0.1021 | 0.1009 | 0.8149 |
| rs544130388 | G | 0.04938 | 0.041 | 4/183/1606 | 0.1021 | 0.1009 | 0.8149 |
| rs564704117 | T | 0.05004 | 0.041 | 4/186/1603 | 0.1037 | 0.1023 | 0.816 |
| rs532179315 | G | 0.04938 | 0.041 | 4/183/1606 | 0.1021 | 0.1009 | 0.8149 |
| rs200905767 | G | 0.04938 | 0.041 | 4/183/1606 | 0.1021 | 0.1009 | 0.8149 |
| rs565742219 | C | 0.04938 | 0.041 | 4/183/1606 | 0.1021 | 0.1009 | 0.8149 |
| rs78064114 | C | 0.04982 | 0.041 | 4/185/1604 | 0.1032 | 0.1018 | 0.8155 |
| rs1554379 | G | 0.4228 | 0.4599 | 339/864/590 | 0.4819 | 0.4902 | 0.4702 |
| rs75895094 | T | 0.05049 | 0.041 | 4/188/1601 | 0.1049 | 0.1033 | 0.8172 |
| rs35653337 | T | 0.05004 | 0.041 | 4/186/1603 | 0.1037 | 0.1023 | 0.816 |
| rs79377543 | G | 0.05004 | 0.041 | 4/186/1603 | 0.1037 | 0.1023 | 0.816 |
| rs75333496 | C | 0.05004 | 0.041 | 4/186/1603 | 0.1037 | 0.1023 | 0.816 |
| rs148303091 | A | 0.0496 | 0.041 | 4/184/1605 | 0.1026 | 0.1013 | 0.8151 |
| rs115912951 | G | 0.0496 | 0.041 | 4/184/1605 | 0.1026 | 0.1013 | 0.8151 |
| rs138430286 | T | 0.0496 | 0.041 | 4/184/1605 | 0.1026 | 0.1013 | 0.8151 |
| rs12625071 | C | 0.0496 | 0.041 | 4/184/1605 | 0.1026 | 0.1013 | 0.8151 |
| rs6053005 | C | 0.4264 | 0.4675 | 347/865/581 | 0.4824 | 0.4915 | 0.4421 |
| rs6053006 | A | 0.3623 | 0.377 | 238/836/719 | 0.4663 | 0.464 | 0.8786 |
| rs144015304 | C | 0.0496 | 0.041 | 4/184/1605 | 0.1026 | 0.1013 | 0.8151 |
| rs115107415 | A | 0.0496 | 0.041 | 4/184/1605 | 0.1026 | 0.1013 | 0.8151 |
| rs76434940 | T | 0.04982 | 0.041 | 4/185/1604 | 0.1032 | 0.1018 | 0.8155 |
| rs79766484 | C | 0.04982 | 0.041 | 4/185/1604 | 0.1032 | 0.1018 | 0.8155 |
| rs79962391 | T | 0.04982 | 0.041 | 4/185/1604 | 0.1032 | 0.1018 | 0.8155 |
| rs6038026 | A | 0.3621 | 0.3841 | 240/832/721 | 0.464 | 0.464 | 1 |
| rs78629244 | A | 0.0496 | 0.04194 | 4/185/1604 | 0.1032 | 0.1018 | 0.8155 |
| rs76835352 | A | 0.0496 | 0.04194 | 4/185/1604 | 0.1032 | 0.1018 | 0.8155 |
| rs2748897 | T | 0.3625 | 0.3817 | 240/834/719 | 0.4651 | 0.4643 | 0.9595 |
| rs2748898 | G | 0.425 | 0.467 | 343/867/583 | 0.4835 | 0.491 | 0.5318 |
| rs12626128 | T | 0.0496 | 0.04194 | 4/185/1604 | 0.1032 | 0.1018 | 0.8155 |
| rs12626067 | C | 0.0496 | 0.04194 | 4/185/1604 | 0.1032 | 0.1018 | 0.8155 |
| rs12625464 | C | 0.0496 | 0.04194 | 4/185/1604 | 0.1032 | 0.1018 | 0.8155 |
| rs12626153 | T | 0.0496 | 0.04194 | 4/185/1604 | 0.1032 | 0.1018 | 0.8155 |
| rs79054518 | C | 0.0496 | 0.04194 | 4/185/1604 | 0.1032 | 0.1018 | 0.8155 |
| rs76164996 | C | 0.0496 | 0.04194 | 4/185/1604 | 0.1032 | 0.1018 | 0.8155 |
| rs74654281 | G | 0.0496 | 0.04194 | 4/185/1604 | 0.1032 | 0.1018 | 0.8155 |
| rs117554664 | G | 0.0496 | 0.04194 | 4/185/1604 | 0.1032 | 0.1018 | 0.8155 |
| rs36107804 | A | 0.02625 | 0.02545 | 1/74/1718 | 0.04127 | 0.04149 | 0.556 |
| rs142637347 | A | 0.0496 | 0.04194 | 4/185/1604 | 0.1032 | 0.1018 | 0.8155 |
| rs150973227 | A | 0.0496 | 0.04194 | 4/185/1604 | 0.1032 | 0.1018 | 0.8155 |
| rs2748899 | C | 0.3612 | 0.385 | 234/839/720 | 0.4679 | 0.4633 | 0.6837 |
| rs72550899 | C | 0.0496 | 0.04194 | 4/185/1604 | 0.1032 | 0.1018 | 0.8155 |
| rs13042023 | T | 0.4086 | 0.4472 | 304/881/608 | 0.4914 | 0.4856 | 0.6271 |
| rs6133182 | T | 0.4084 | 0.4449 | 309/866/618 | 0.483 | 0.4852 | 0.8457 |
| rs6053010 | C | 0.02335 | 0.0132 | 1/82/1710 | 0.04573 | 0.04575 | 1 |
| rs6053011 | C | 0.5169 | 0.4557 | 463/906/424 | 0.5053 | 0.4998 | 0.6706 |
| rs6053012 | C | 0.5169 | 0.4557 | 463/906/424 | 0.5053 | 0.4998 | 0.6706 |
| rs13042159 | G | 0.01535 | 0.009896 | 1/51/1741 | 0.02844 | 0.02912 | 0.3229 |
| rs6053013 | T | 0.0109 | 0.01697 | 1/41/1751 | 0.02287 | 0.02369 | 0.225 |
| rs4813726 | G | 0.4573 | 0.3633 | 382/892/519 | 0.4975 | 0.4971 | 1 |
| rs1105838 | C | 0.4468 | 0.3516 | 364/891/538 | 0.4969 | 0.4953 | 0.924 |
| rs2203907 | G | 0.4895 | 0.5429 | 431/899/463 | 0.5014 | 0.4998 | 0.9247 |
| rs4076098 | G | 0.4464 | 0.3516 | 364/889/540 | 0.4958 | 0.4952 | 1 |
| rs2326576 | T | 0.4566 | 0.3633 | 382/890/521 | 0.4964 | 0.497 | 0.9621 |
| rs2681118 | G | 0.01112 | 0.01791 | 1/42/1750 | 0.02342 | 0.02424 | 0.2344 |
| rs13044890 | C | 0.4455 | 0.3511 | 364/887/542 | 0.4947 | 0.4951 | 1 |
| rs111733047 | T | 0.2375 | 0.3087 | 101/617/1075 | 0.3441 | 0.3525 | 0.3151 |
| rs2203908 | A | 0.4457 | 0.3516 | 364/887/542 | 0.4947 | 0.4951 | 1 |
| rs1923094 | G | 0.3147 | 0.3256 | 194/761/838 | 0.4244 | 0.4355 | 0.2785 |
| rs6116605 | T | 0.3112 | 0.3214 | 194/748/851 | 0.4172 | 0.4329 | 0.1267 |
| rs2064842 | C | 0.01112 | 0.01791 | 0/43/1750 | 0.02398 | 0.02369 | 1 |
| rs6116606 | A | 0.3112 | 0.3214 | 194/748/851 | 0.4172 | 0.4329 | 0.1267 |
| rs6053016 | C | 0.3123 | 0.3214 | 196/748/849 | 0.4172 | 0.4337 | 0.1142 |
| rs6053017 | G | 0.3112 | 0.3214 | 194/748/851 | 0.4172 | 0.4329 | 0.1267 |
| rs6053018 | C | 0.3116 | 0.3219 | 195/747/851 | 0.4166 | 0.4331 | 0.1137 |
| rs6053019 | C | 0.3112 | 0.3214 | 194/748/851 | 0.4172 | 0.4329 | 0.1267 |
| rs6053020 | G | 0.3123 | 0.3214 | 196/748/849 | 0.4172 | 0.4337 | 0.1142 |
| rs6053021 | T | 0.3109 | 0.3214 | 194/747/852 | 0.4166 | 0.4327 | 0.1138 |
| rs1969715 | G | 0.3109 | 0.3214 | 194/747/852 | 0.4166 | 0.4327 | 0.1138 |
| rs113321431 | A | 0.3116 | 0.3214 | 195/748/850 | 0.4172 | 0.4333 | 0.1142 |
| rs6053022 | G | 0.3129 | 0.3214 | 197/749/847 | 0.4177 | 0.4343 | 0.1147 |
| rs6139598 | T | 0.3109 | 0.3214 | 194/747/852 | 0.4166 | 0.4327 | 0.1138 |
| rs1971573 | C | 0.3356 | 0.3487 | 227/774/792 | 0.4317 | 0.4504 | 0.08346 |
| rs1543452 | T | 0.3109 | 0.3214 | 194/747/852 | 0.4166 | 0.4327 | 0.1138 |
| rs1519861 | A | 0.3109 | 0.3214 | 194/747/852 | 0.4166 | 0.4327 | 0.1138 |
| rs2423076 | T | 0.3029 | 0.3318 | 168/749/876 | 0.4177 | 0.422 | 0.6548 |
| rs58235874 | A | 0.01112 | 0.01791 | 0/43/1750 | 0.02398 | 0.02369 | 1 |
| rs6139600 | A | 0.3127 | 0.3228 | 197/748/848 | 0.4172 | 0.4341 | 0.1026 |
| rs6139601 | A | 0.3141 | 0.3233 | 199/749/845 | 0.4177 | 0.4351 | 0.09251 |
| rs6133183 | C | 0.3127 | 0.3228 | 197/748/848 | 0.4172 | 0.4341 | 0.1026 |
| rs6053024 | T | 0.3129 | 0.3228 | 197/749/847 | 0.4177 | 0.4343 | 0.1147 |
| rs62200399 | A | 0.04026 | 0.00754 | 4/158/1631 | 0.08812 | 0.0883 | 0.7904 |
| rs6139606 | G | 0.3112 | 0.3172 | 194/743/856 | 0.4144 | 0.4318 | 0.08999 |
| rs1401828 | T | 0.3087 | 0.3134 | 194/736/863 | 0.4105 | 0.4304 | 0.05467 |
| rs4815759 | A | 0.3076 | 0.3124 | 192/739/862 | 0.4122 | 0.4302 | 0.07892 |
| rs1279682 | T | 0.01379 | 0.01838 | 1/51/1741 | 0.02844 | 0.02912 | 0.3229 |
| rs6084957 | T | 0.3072 | 0.3124 | 190/742/861 | 0.4138 | 0.43 | 0.1114 |
| rs12479919 | T | 0.3069 | 0.3129 | 190/741/862 | 0.4133 | 0.4298 | 0.1109 |
| rs13042903 | A | 0.3067 | 0.3129 | 189/742/862 | 0.4138 | 0.4296 | 0.1237 |
| rs113708270 | G | 0.3863 | 0.3497 | 264/836/693 | 0.4663 | 0.4714 | 0.6521 |
| rs2423082 | G | 0.02669 | 0.0278 | 1/100/1692 | 0.05577 | 0.05527 | 1 |
| rs2423083 | G | 0.01557 | 0.01885 | 0/61/1732 | 0.03402 | 0.03344 | 1 |
| rs2254964 | G | 0.01557 | 0.01885 | 0/61/1732 | 0.03402 | 0.03344 | 1 |
| rs2423084 | C | 0.01557 | 0.01885 | 0/61/1732 | 0.03402 | 0.03344 | 1 |
| rs1279683 | A | 0.377 | 0.3407 | 248/835/710 | 0.4657 | 0.4668 | 0.9195 |
| rs55853468 | G | 0.3601 | 0.3219 | 235/794/764 | 0.4428 | 0.4565 | 0.2143 |
| rs2423085 | T | 0.0149 | 0.01838 | 0/58/1735 | 0.03235 | 0.03182 | 1 |
| rs6053029 | G | 0.2473 | 0.3266 | 113/630/1050 | 0.3514 | 0.3635 | 0.1721 |
| rs6139609 | A | 0.3592 | 0.3214 | 235/790/768 | 0.4406 | 0.4558 | 0.1618 |
| rs6053030 | T | 0.3763 | 0.3407 | 247/837/709 | 0.4668 | 0.4668 | 1 |
| rs6133184 | T | 0.3592 | 0.3214 | 235/790/768 | 0.4406 | 0.4558 | 0.1618 |
| rs2423086 | G | 0.0149 | 0.01838 | 0/58/1735 | 0.03235 | 0.03182 | 1 |
| rs6038038 | T | 0.01379 | 0.01791 | 0/54/1739 | 0.03012 | 0.02966 | 1 |

NOTE:SNP, Single Nucleotide Polymorphism;A1,minor allele; MAF, Minor allele frequency; NSCLO,Non-syndromic cleft lip only; GENO, Genotype counts; O(HET), Observed heterozygosity; E(HET), Expected heterozygosity; P, Hardy-Weinberg test p value.
