# Supplementary material for: Oxidative stress-mediated apoptosis via the SLC23A2-ascorbic acid interaction contributes to cleft lip development
Source: Front Pediatr. 2025 Oct 2;13:1632778. doi: 10.3389/fped.2025.1632778 (PMC12527864; doi:10.3389/fped.2025.1632778)
Supplement: Supplementary file 3 [file Table3.docx]

**Appendix Table 3** Allelic association analysis of SNPs at SLC23A2 in NSCLO cases and controls

| CHR | SNP | BP | A1 | OR | L95 | U95 | P |
| --- | --- | --- | --- | --- | --- | --- | --- |
| 20 | rs6053029 | 4984381 | G | 1.888 | 1.625 | 2.194 | 9.44E-17 |
| 20 | rs62200399 | 4976240 | A | 0.09765 | 0.05613 | 0.1699 | 1.81E-16 |
| 20 | rs1105838 | 4967199 | C | 0.573 | 0.501 | 0.6553 | 4.35E-16 |
| 20 | rs13044890 | 4968220 | C | 0.5741 | 0.502 | 0.6566 | 5.27E-16 |
| 20 | rs4076098 | 4967355 | G | 0.5743 | 0.5022 | 0.6568 | 5.52E-16 |
| 20 | rs2203908 | 4969110 | A | 0.5752 | 0.503 | 0.6577 | 6.19E-16 |
| 20 | rs111733047 | 4968310 | T | 1.869 | 1.605 | 2.177 | 9.07E-16 |
| 20 | rs4813726 | 4966941 | G | 0.5883 | 0.515 | 0.672 | 5.66E-15 |
| 20 | rs2326576 | 4967393 | T | 0.5897 | 0.5162 | 0.6736 | 7.20E-15 |
| 20 | rs36107804 | 4962438 | A | 3.464 | 2.132 | 5.627 | 5.19E-07 |
| 20 | rs2203907 | 4967335 | C | 0.7895 | 0.6927 | 0.8999 | 0.0003994 |
| 20 | rs113678532 | 4937733 | A | 0.3958 | 0.2134 | 0.7341 | 0.003277 |
| 20 | rs7260796 | 4936110 | C | 0.4216 | 0.2302 | 0.7722 | 0.005153 |
| 20 | rs57215863 | 4936293 | T | 0.4216 | 0.2302 | 0.7722 | 0.005153 |
| 20 | rs16990455 | 4936526 | T | 0.4216 | 0.2302 | 0.7722 | 0.005153 |
| 20 | rs75511237 | 4939377 | C | 0.4156 | 0.2201 | 0.7846 | 0.006762 |
| 20 | rs79494287 | 4937244 | A | 0.4436 | 0.2375 | 0.8287 | 0.01079 |
| 20 | rs111806610 | 4925327 | A | 0.4777 | 0.2633 | 0.8667 | 0.01507 |
| 20 | rs6053011 | 4964118 | C | 0.8514 | 0.7466 | 0.971 | 0.01642 |
| 20 | rs6053012 | 4964119 | C | 0.8514 | 0.7466 | 0.971 | 0.01642 |
| 20 | rs6053030 | 4985342 | T | 0.8669 | 0.7586 | 0.9906 | 0.0358 |
| 20 | rs6139609 | 4984984 | A | 0.8669 | 0.7581 | 0.9912 | 0.03665 |
| 20 | rs6133184 | 4985373 | T | 0.8669 | 0.7581 | 0.9912 | 0.03665 |
| 20 | rs2748901 | 4948248 | A | 1.153 | 1.009 | 1.318 | 0.037 |
| 20 | rs1279683 | 4983092 | A | 0.8679 | 0.7596 | 0.9917 | 0.03724 |
| 20 | rs55853468 | 4983318 | G | 0.868 | 0.7591 | 0.9925 | 0.03846 |
| 20 | rs2681116 | 4951331 | C | 1.144 | 1.003 | 1.304 | 0.04534 |
| 20 | rs113708270 | 4982118 | G | 0.8748 | 0.7665 | 0.9983 | 0.04712 |
| 20 | rs190393511 | 4941761 | C | 0.578 | 0.325 | 1.028 | 0.06196 |
| 20 | rs2748898 | 4961229 | G | 1.132 | 0.9927 | 1.29 | 0.06416 |
| 20 | rs2423076 | 4974608 | T | 1.14 | 0.992 | 1.31 | 0.06485 |
| 20 | rs6053005 | 4957700 | C | 1.131 | 0.9924 | 1.29 | 0.06496 |
| 20 | rs6139592 | 4952773 | A | 1.13 | 0.9907 | 1.289 | 0.06861 |
| 20 | rs1554379 | 4953680 | G | 1.13 | 0.9904 | 1.289 | 0.06932 |
| 20 | rs6139593 | 4952923 | T | 1.129 | 0.9901 | 1.288 | 0.07001 |
| 20 | rs6053003 | 4953288 | A | 1.129 | 0.9901 | 1.288 | 0.07001 |
| 20 | rs13037458 | 4951215 | C | 1.129 | 0.9896 | 1.287 | 0.07124 |
| 20 | rs7263163 | 4952586 | T | 1.127 | 0.9878 | 1.285 | 0.07557 |
| 20 | rs6053010 | 4964103 | C | 0.6536 | 0.386 | 1.107 | 0.1135 |
| 20 | rs6107559 | 4941838 | G | 1.11 | 0.9713 | 1.269 | 0.1251 |
| 20 | rs75895094 | 4953858 | T | 0.8137 | 0.5959 | 1.111 | 0.1948 |
| 20 | rs35731094 | 4951315 | A | 0.8142 | 0.5962 | 1.112 | 0.1962 |
| 20 | rs114776261 | 4951919 | T | 0.8142 | 0.5962 | 1.112 | 0.1962 |
| 20 | rs187777103 | 4952294 | T | 0.8142 | 0.5962 | 1.112 | 0.1962 |
| 20 | rs201341095 | 4952295 | C | 0.8142 | 0.5962 | 1.112 | 0.1962 |
| 20 | rs115233603 | 4952556 | T | 0.8142 | 0.5962 | 1.112 | 0.1962 |
| 20 | rs76480995 | 4952679 | T | 0.8142 | 0.5962 | 1.112 | 0.1962 |
| 20 | rs79774064 | 4952874 | C | 0.8146 | 0.5965 | 1.113 | 0.1974 |
| 20 | rs192759748 | 4953222 | T | 0.8146 | 0.5965 | 1.113 | 0.1974 |
| 20 | rs146463412 | 4953388 | C | 0.8146 | 0.5965 | 1.113 | 0.1974 |
| 20 | rs542764191 | 4953482 | T | 0.8146 | 0.5965 | 1.113 | 0.1974 |
| 20 | rs564704117 | 4953511 | T | 0.8146 | 0.5965 | 1.113 | 0.1974 |
| 20 | rs35653337 | 4954186 | T | 0.8146 | 0.5965 | 1.113 | 0.1974 |
| 20 | rs79377543 | 4954439 | G | 0.8146 | 0.5965 | 1.113 | 0.1974 |
| 20 | rs75333496 | 4954665 | C | 0.8146 | 0.5965 | 1.113 | 0.1974 |
| 20 | rs6133182 | 4964088 | T | 1.09 | 0.9556 | 1.244 | 0.1989 |
| 20 | rs76434940 | 4958588 | T | 0.8154 | 0.597 | 1.114 | 0.1996 |
| 20 | rs79766484 | 4959074 | C | 0.8154 | 0.597 | 1.114 | 0.1996 |
| 20 | rs79962391 | 4959167 | T | 0.8154 | 0.597 | 1.114 | 0.1996 |
| 20 | rs148303091 | 4955606 | A | 0.816 | 0.5973 | 1.115 | 0.2014 |
| 20 | rs115912951 | 4956446 | G | 0.816 | 0.5973 | 1.115 | 0.2014 |
| 20 | rs138430286 | 4956600 | T | 0.816 | 0.5973 | 1.115 | 0.2014 |
| 20 | rs12625071 | 4957608 | C | 0.816 | 0.5973 | 1.115 | 0.2014 |
| 20 | rs144015304 | 4958131 | C | 0.816 | 0.5973 | 1.115 | 0.2014 |
| 20 | rs115107415 | 4958273 | A | 0.816 | 0.5973 | 1.115 | 0.2014 |
| 20 | rs3787456 | 4851901 | A | 0.807 | 0.5724 | 1.138 | 0.221 |
| 20 | rs577998756 | 4953462 | T | 0.8228 | 0.6018 | 1.125 | 0.2218 |
| 20 | rs545078767 | 4953464 | C | 0.8228 | 0.6018 | 1.125 | 0.2218 |
| 20 | rs1776952 | 4934502 | T | 0.8499 | 0.6506 | 1.11 | 0.2331 |
| 20 | rs78064114 | 4953542 | C | 0.8274 | 0.6055 | 1.13 | 0.2341 |
| 20 | rs78629244 | 4960596 | A | 0.8313 | 0.6105 | 1.132 | 0.241 |
| 20 | rs76835352 | 4960933 | A | 0.8313 | 0.6105 | 1.132 | 0.241 |
| 20 | rs12626128 | 4961246 | T | 0.8313 | 0.6105 | 1.132 | 0.241 |
| 20 | rs12626067 | 4961272 | C | 0.8313 | 0.6105 | 1.132 | 0.241 |
| 20 | rs12625464 | 4961380 | C | 0.8313 | 0.6105 | 1.132 | 0.241 |
| 20 | rs12626153 | 4961407 | T | 0.8313 | 0.6105 | 1.132 | 0.241 |
| 20 | rs79054518 | 4961460 | C | 0.8313 | 0.6105 | 1.132 | 0.241 |
| 20 | rs76164996 | 4961634 | C | 0.8313 | 0.6105 | 1.132 | 0.241 |
| 20 | rs74654281 | 4962021 | G | 0.8313 | 0.6105 | 1.132 | 0.241 |
| 20 | rs117554664 | 4962379 | G | 0.8313 | 0.6105 | 1.132 | 0.241 |
| 20 | rs142637347 | 4962549 | A | 0.8313 | 0.6105 | 1.132 | 0.241 |
| 20 | rs150973227 | 4962599 | A | 0.8313 | 0.6105 | 1.132 | 0.241 |
| 20 | rs72550899 | 4963412 | C | 0.8313 | 0.6105 | 1.132 | 0.241 |
| 20 | rs1715397 | 4933888 | G | 0.8549 | 0.6542 | 1.117 | 0.2506 |
| 20 | rs1715361 | 4934540 | A | 0.8549 | 0.6542 | 1.117 | 0.2506 |
| 20 | rs553897675 | 4953477 | T | 0.8329 | 0.6087 | 1.14 | 0.2531 |
| 20 | rs572298297 | 4953478 | A | 0.8329 | 0.6087 | 1.14 | 0.2531 |
| 20 | rs192725418 | 4953495 | A | 0.8374 | 0.6124 | 1.145 | 0.2665 |
| 20 | rs531873467 | 4953509 | T | 0.8374 | 0.6124 | 1.145 | 0.2665 |
| 20 | rs544130388 | 4953510 | G | 0.8374 | 0.6124 | 1.145 | 0.2665 |
| 20 | rs532179315 | 4953512 | G | 0.8374 | 0.6124 | 1.145 | 0.2665 |
| 20 | rs200905767 | 4953518 | G | 0.8374 | 0.6124 | 1.145 | 0.2665 |
| 20 | rs565742219 | 4953519 | C | 0.8374 | 0.6124 | 1.145 | 0.2665 |
| 20 | rs1715392 | 4925577 | T | 0.8599 | 0.6581 | 1.124 | 0.2686 |
| 20 | rs1519862 | 4927354 | A | 0.8599 | 0.6581 | 1.124 | 0.2686 |
| 20 | rs1519865 | 4931116 | A | 0.8655 | 0.6622 | 1.131 | 0.2902 |
| 20 | rs1715395 | 4931592 | G | 0.8655 | 0.6622 | 1.131 | 0.2902 |
| 20 | rs1776950 | 4932698 | A | 0.8655 | 0.6622 | 1.131 | 0.2902 |
| 20 | rs1776947 | 4926855 | G | 0.8657 | 0.6624 | 1.131 | 0.2911 |
| 20 | rs1519864 | 4927886 | G | 0.8662 | 0.6627 | 1.132 | 0.293 |
| 20 | rs113257858 | 4924164 | T | 0.8668 | 0.6631 | 1.133 | 0.2954 |
| 20 | rs13042023 | 4964050 | T | 1.071 | 0.9384 | 1.223 | 0.3086 |
| 20 | rs1776967 | 4891536 | T | 0.782 | 0.4862 | 1.258 | 0.3105 |
| 20 | rs1715381 | 4883553 | T | 0.9303 | 0.8033 | 1.077 | 0.3348 |
| 20 | rs1715386 | 4887900 | T | 0.9038 | 0.7351 | 1.111 | 0.3374 |
| 20 | rs1715384 | 4884978 | T | 0.9309 | 0.8041 | 1.078 | 0.3379 |
| 20 | rs79137959 | 4835665 | C | 1.146 | 0.8659 | 1.517 | 0.3406 |
| 20 | rs4813723 | 4887784 | C | 0.9325 | 0.8073 | 1.077 | 0.3424 |
| 20 | rs3914810 | 4927713 | C | 0.931 | 0.8021 | 1.081 | 0.3474 |
| 20 | rs6116569 | 4864717 | C | 0.9334 | 0.8058 | 1.081 | 0.3578 |
| 20 | rs2748896 | 4885110 | A | 0.9194 | 0.7684 | 1.1 | 0.3582 |
| 20 | rs4987219 | 4864946 | G | 0.9348 | 0.8048 | 1.086 | 0.3775 |
| 20 | rs1715385 | 4887670 | G | 0.9373 | 0.8105 | 1.084 | 0.383 |
| 20 | rs2681110 | 4941884 | T | 1.061 | 0.929 | 1.211 | 0.3837 |
| 20 | rs6038010 | 4863786 | C | 0.9372 | 0.807 | 1.089 | 0.3959 |
| 20 | rs1715383 | 4884728 | G | 0.9264 | 0.7726 | 1.111 | 0.4094 |
| 20 | rs1715379 | 4879927 | G | 0.9415 | 0.8136 | 1.089 | 0.418 |
| 20 | rs1776964 | 4880308 | G | 0.9416 | 0.8137 | 1.09 | 0.4189 |
| 20 | rs6038007 | 4855759 | T | 0.9415 | 0.8107 | 1.093 | 0.4299 |
| 20 | rs1776959 | 4871605 | G | 0.9422 | 0.8124 | 1.093 | 0.4309 |
| 20 | rs8122118 | 4855880 | T | 0.9425 | 0.8097 | 1.097 | 0.445 |
| 20 | rs12329577 | 4847010 | G | 0.9336 | 0.7804 | 1.117 | 0.4528 |
| 20 | rs8115867 | 4855678 | G | 0.9439 | 0.8111 | 1.098 | 0.4555 |
| 20 | rs1715380 | 4881960 | C | 0.9328 | 0.7768 | 1.12 | 0.4563 |
| 20 | rs2748899 | 4963241 | C | 1.052 | 0.9195 | 1.204 | 0.4599 |
| 20 | rs6038026 | 4959654 | A | 1.052 | 0.9194 | 1.204 | 0.4611 |
| 20 | rs1776965 | 4883727 | T | 0.9339 | 0.7766 | 1.123 | 0.4671 |
| 20 | rs1776960 | 4871964 | T | 0.9465 | 0.816 | 1.098 | 0.4674 |
| 20 | rs3787459 | 4857357 | A | 0.9453 | 0.8112 | 1.101 | 0.4707 |
| 20 | rs1776963 | 4878981 | T | 0.9401 | 0.7944 | 1.113 | 0.4726 |
| 20 | rs1614554 | 4894220 | C | 0.8395 | 0.5199 | 1.356 | 0.4743 |
| 20 | rs1776970 | 4895761 | C | 0.8395 | 0.5199 | 1.356 | 0.4743 |
| 20 | rs1715362 | 4895803 | T | 0.8395 | 0.5199 | 1.356 | 0.4743 |
| 20 | rs8123436 | 4856089 | T | 0.946 | 0.8123 | 1.102 | 0.4747 |
| 20 | rs1715387 | 4889609 | T | 0.8402 | 0.5202 | 1.357 | 0.4764 |
| 20 | rs4815754 | 4886490 | A | 0.9288 | 0.7572 | 1.139 | 0.4786 |
| 20 | rs3787458 | 4856192 | G | 0.9483 | 0.8152 | 1.103 | 0.4917 |
| 20 | rs1776955 | 4868309 | C | 0.9516 | 0.8254 | 1.097 | 0.4943 |
| 20 | rs3787457 | 4856160 | T | 0.9492 | 0.8153 | 1.105 | 0.5015 |
| 20 | rs6052956 | 4868291 | T | 0.9497 | 0.8171 | 1.104 | 0.5016 |
| 20 | rs13045713 | 4847211 | G | 0.9446 | 0.7999 | 1.115 | 0.5018 |
| 20 | rs2681113 | 4947430 | C | 1.047 | 0.9147 | 1.198 | 0.5071 |
| 20 | rs79181767 | 4867534 | C | 0.8689 | 0.5683 | 1.329 | 0.5166 |
| 20 | rs1715368 | 4903042 | C | 0.8579 | 0.5336 | 1.38 | 0.5272 |
| 20 | rs74912238 | 4903924 | A | 0.8579 | 0.5336 | 1.38 | 0.5272 |
| 20 | rs73897144 | 4903925 | T | 0.8579 | 0.5336 | 1.38 | 0.5272 |
| 20 | rs2946682 | 4915298 | G | 0.9219 | 0.7157 | 1.188 | 0.529 |
| 20 | rs1343095 | 4917363 | G | 0.9219 | 0.7157 | 1.188 | 0.529 |
| 20 | rs2298174 | 4864570 | C | 0.9533 | 0.818 | 1.111 | 0.5403 |
| 20 | rs2748902 | 4946471 | G | 1.042 | 0.9107 | 1.192 | 0.5489 |
| 20 | rs1715377 | 4879297 | A | 0.9427 | 0.7767 | 1.144 | 0.5502 |
| 20 | rs2748897 | 4961077 | T | 1.041 | 0.91 | 1.192 | 0.5556 |
| 20 | rs138782359 | 4911357 | T | 0.9277 | 0.7197 | 1.196 | 0.5621 |
| 20 | rs77395977 | 4883736 | T | 0.9416 | 0.7676 | 1.155 | 0.5634 |
| 20 | rs1715382 | 4883803 | C | 0.9416 | 0.7676 | 1.155 | 0.5634 |
| 20 | rs6052961 | 4878673 | T | 0.9263 | 0.7142 | 1.201 | 0.5636 |
| 20 | rs6052962 | 4882222 | A | 0.8744 | 0.5469 | 1.398 | 0.5751 |
| 20 | rs1776966 | 4887198 | A | 0.9422 | 0.7618 | 1.165 | 0.5826 |
| 20 | rs6052988 | 4942600 | C | 1.039 | 0.9073 | 1.189 | 0.5829 |
| 20 | rs2681118 | 4968002 | G | 1.155 | 0.6908 | 1.93 | 0.5834 |
| 20 | rs74333140 | 4887934 | C | 0.8757 | 0.5435 | 1.411 | 0.5854 |
| 20 | rs6084944 | 4942200 | A | 1.037 | 0.9065 | 1.187 | 0.5938 |
| 20 | rs76415638 | 4906156 | T | 0.9224 | 0.6851 | 1.242 | 0.5946 |
| 20 | rs189470744 | 4915827 | T | 0.9224 | 0.6851 | 1.242 | 0.5946 |
| 20 | rs939258 | 4906841 | A | 0.9344 | 0.7254 | 1.204 | 0.5994 |
| 20 | rs1776958 | 4870997 | G | 0.9627 | 0.8318 | 1.114 | 0.6095 |
| 20 | rs1891649 | 4853932 | A | 0.9618 | 0.826 | 1.12 | 0.6161 |
| 20 | rs146487465 | 4850136 | C | 1.082 | 0.7913 | 1.48 | 0.6213 |
| 20 | rs13037855 | 4921733 | T | 1.113 | 0.7257 | 1.707 | 0.6239 |
| 20 | rs78624304 | 4859126 | T | 1.07 | 0.8168 | 1.401 | 0.6248 |
| 20 | rs77881678 | 4890675 | T | 0.8882 | 0.5498 | 1.435 | 0.6281 |
| 20 | rs1715374 | 4870044 | G | 0.9642 | 0.8314 | 1.118 | 0.63 |
| 20 | rs80244584 | 4905666 | G | 0.9309 | 0.6923 | 1.252 | 0.6354 |
| 20 | rs12481275 | 4862479 | A | 0.9643 | 0.8299 | 1.121 | 0.6356 |
| 20 | rs6139587 | 4942474 | T | 1.033 | 0.9022 | 1.183 | 0.6388 |
| 20 | rs6139606 | 4977465 | G | 1.033 | 0.8986 | 1.188 | 0.6452 |
| 20 | rs35560557 | 4834894 | A | 1.047 | 0.8587 | 1.278 | 0.6476 |
| 20 | rs6053013 | 4964516 | T | 1.128 | 0.6694 | 1.901 | 0.6509 |
| 20 | rs2681109 | 4937745 | A | 1.081 | 0.7702 | 1.518 | 0.652 |
| 20 | rs12480138 | 4953167 | G | 1.031 | 0.9005 | 1.18 | 0.6585 |
| 20 | rs12481301 | 4953175 | T | 1.031 | 0.9005 | 1.18 | 0.6585 |
| 20 | rs1776977 | 4909220 | T | 0.8995 | 0.5616 | 1.441 | 0.6595 |
| 20 | rs1776978 | 4913708 | A | 0.8995 | 0.5616 | 1.441 | 0.6595 |
| 20 | rs939259 | 4906843 | A | 0.9002 | 0.5619 | 1.442 | 0.662 |
| 20 | rs1715370 | 4908569 | T | 0.9002 | 0.5619 | 1.442 | 0.662 |
| 20 | rs1628664 | 4914720 | A | 0.9002 | 0.5619 | 1.442 | 0.662 |
| 20 | rs6139600 | 4975304 | A | 1.031 | 0.8975 | 1.185 | 0.6649 |
| 20 | rs6133183 | 4975431 | C | 1.031 | 0.8975 | 1.185 | 0.6649 |
| 20 | rs1715372 | 4910589 | G | 1.042 | 0.8659 | 1.253 | 0.665 |
| 20 | rs6053024 | 4975805 | T | 1.031 | 0.8973 | 1.185 | 0.667 |
| 20 | rs6053021 | 4972509 | T | 1.031 | 0.8972 | 1.185 | 0.6678 |
| 20 | rs1969715 | 4972688 | G | 1.031 | 0.8972 | 1.185 | 0.6678 |
| 20 | rs6139598 | 4973740 | T | 1.031 | 0.8972 | 1.185 | 0.6678 |
| 20 | rs1543452 | 4974106 | T | 1.031 | 0.8972 | 1.185 | 0.6678 |
| 20 | rs1519861 | 4974601 | A | 1.031 | 0.8972 | 1.185 | 0.6678 |
| 20 | rs1715373 | 4867140 | G | 0.9694 | 0.8377 | 1.122 | 0.6769 |
| 20 | rs1715360 | 4916369 | C | 0.9053 | 0.566 | 1.448 | 0.6781 |
| 20 | rs6053018 | 4971886 | C | 1.03 | 0.8962 | 1.183 | 0.6798 |
| 20 | rs1776953 | 4941291 | T | 1.102 | 0.6945 | 1.749 | 0.68 |
| 20 | rs181793493 | 4915828 | A | 0.906 | 0.5663 | 1.449 | 0.6806 |
| 20 | rs117968930 | 4905277 | G | 0.9394 | 0.6976 | 1.265 | 0.6807 |
| 20 | rs73893863 | 4913904 | A | 0.9396 | 0.6984 | 1.264 | 0.6809 |
| 20 | rs6139601 | 4975407 | A | 1.03 | 0.896 | 1.183 | 0.6814 |
| 20 | rs58061452 | 4953230 | T | 1.028 | 0.8982 | 1.178 | 0.685 |
| 20 | rs1629176 | 4869790 | A | 0.9698 | 0.8361 | 1.125 | 0.6857 |
| 20 | rs6139591 | 4951359 | A | 1.028 | 0.8979 | 1.177 | 0.6877 |
| 20 | rs6053002 | 4951928 | C | 1.028 | 0.8979 | 1.177 | 0.6877 |
| 20 | rs6116605 | 4970113 | T | 1.029 | 0.8954 | 1.182 | 0.6877 |
| 20 | rs6116606 | 4971299 | A | 1.029 | 0.8954 | 1.182 | 0.6877 |
| 20 | rs6053017 | 4971825 | G | 1.029 | 0.8954 | 1.182 | 0.6877 |
| 20 | rs6053019 | 4972062 | C | 1.029 | 0.8954 | 1.182 | 0.6877 |
| 20 | rs113321431 | 4973446 | A | 1.029 | 0.8954 | 1.182 | 0.6884 |
| 20 | rs1131382 | 4834338 | C | 0.9739 | 0.8555 | 1.109 | 0.6892 |
| 20 | rs73894175 | 4905262 | G | 0.9412 | 0.6994 | 1.267 | 0.6893 |
| 20 | rs56671692 | 4912279 | A | 0.9412 | 0.6994 | 1.267 | 0.6893 |
| 20 | rs59971708 | 4912308 | A | 0.9412 | 0.6994 | 1.267 | 0.6893 |
| 20 | rs3761240 | 4914872 | A | 0.9412 | 0.6994 | 1.267 | 0.6893 |
| 20 | rs55793575 | 4915790 | A | 0.9412 | 0.6994 | 1.267 | 0.6893 |
| 20 | rs1776956 | 4870126 | G | 0.9706 | 0.837 | 1.125 | 0.6925 |
| 20 | rs1715375 | 4870770 | G | 0.9706 | 0.837 | 1.125 | 0.6925 |
| 20 | rs1971573 | 4973886 | C | 1.028 | 0.8971 | 1.177 | 0.6937 |
| 20 | rs2064842 | 4970721 | C | 1.107 | 0.6635 | 1.848 | 0.6962 |
| 20 | rs58235874 | 4975023 | A | 1.107 | 0.6635 | 1.848 | 0.6962 |
| 20 | rs6053016 | 4971502 | C | 1.028 | 0.8946 | 1.181 | 0.697 |
| 20 | rs6053020 | 4972112 | G | 1.028 | 0.8946 | 1.181 | 0.697 |
| 20 | rs1879177 | 4948435 | T | 1.027 | 0.897 | 1.176 | 0.6996 |
| 20 | rs6052990 | 4943695 | C | 1.027 | 0.8968 | 1.176 | 0.7018 |
| 20 | rs6038021 | 4943696 | A | 1.027 | 0.8968 | 1.176 | 0.7018 |
| 20 | rs6052991 | 4944263 | G | 1.027 | 0.8968 | 1.176 | 0.7018 |
| 20 | rs6052992 | 4944442 | C | 1.027 | 0.8968 | 1.176 | 0.7018 |
| 20 | rs6139588 | 4944510 | A | 1.027 | 0.8968 | 1.176 | 0.7018 |
| 20 | rs6052998 | 4947637 | A | 1.027 | 0.8968 | 1.176 | 0.7018 |
| 20 | rs1879176 | 4948389 | C | 1.027 | 0.8968 | 1.176 | 0.7018 |
| 20 | rs116915305 | 4909636 | A | 0.9441 | 0.7014 | 1.271 | 0.7041 |
| 20 | rs6053006 | 4958064 | A | 1.027 | 0.8965 | 1.176 | 0.7042 |
| 20 | rs1776957 | 4870393 | T | 0.9721 | 0.8384 | 1.127 | 0.7077 |
| 20 | rs6053022 | 4973456 | G | 1.026 | 0.8928 | 1.179 | 0.7178 |
| 20 | rs1110277 | 4854682 | G | 0.9727 | 0.8371 | 1.13 | 0.7181 |
| 20 | rs6038025 | 4949523 | T | 1.025 | 0.8952 | 1.174 | 0.719 |
| 20 | rs6038020 | 4943441 | T | 1.025 | 0.8948 | 1.173 | 0.7248 |
| 20 | rs6038022 | 4945432 | T | 1.025 | 0.8948 | 1.173 | 0.7248 |
| 20 | rs6038038 | 4988076 | T | 1.092 | 0.6614 | 1.802 | 0.7317 |
| 20 | rs939257 | 4906679 | C | 0.9227 | 0.5791 | 1.47 | 0.7349 |
| 20 | rs3787469 | 4902768 | C | 0.95 | 0.7049 | 1.28 | 0.7363 |
| 20 | rs1923094 | 4969239 | G | 1.024 | 0.8914 | 1.177 | 0.7367 |
| 20 | rs1776971 | 4905610 | C | 0.9234 | 0.5795 | 1.471 | 0.7375 |
| 20 | rs1776972 | 4906003 | C | 0.9234 | 0.5795 | 1.471 | 0.7375 |
| 20 | rs1776973 | 4906616 | C | 0.9234 | 0.5795 | 1.471 | 0.7375 |
| 20 | rs2423083 | 4982748 | G | 1.086 | 0.6706 | 1.758 | 0.7377 |
| 20 | rs2254964 | 4982772 | G | 1.086 | 0.6706 | 1.758 | 0.7377 |
| 20 | rs2423084 | 4983084 | C | 1.086 | 0.6706 | 1.758 | 0.7377 |
| 20 | rs1715376 | 4872886 | C | 0.9756 | 0.8416 | 1.131 | 0.7436 |
| 20 | rs6038023 | 4946170 | A | 1.022 | 0.8928 | 1.17 | 0.75 |
| 20 | rs1519866 | 4950259 | G | 1.022 | 0.8927 | 1.171 | 0.7506 |
| 20 | rs6038002 | 4846619 | C | 0.976 | 0.84 | 1.134 | 0.7507 |
| 20 | rs2423085 | 4983822 | T | 1.083 | 0.658 | 1.781 | 0.7549 |
| 20 | rs2423086 | 4986033 | G | 1.083 | 0.658 | 1.781 | 0.7549 |
| 20 | rs1776961 | 4873965 | C | 0.9096 | 0.4933 | 1.677 | 0.7615 |
| 20 | rs12625783 | 4929930 | C | 1.083 | 0.6287 | 1.866 | 0.7733 |
| 20 | rs1401828 | 4977788 | T | 1.021 | 0.8874 | 1.174 | 0.7758 |
| 20 | rs8125856 | 4885829 | T | 1.031 | 0.8217 | 1.294 | 0.7914 |
| 20 | rs1279682 | 4980405 | T | 1.064 | 0.6491 | 1.745 | 0.8049 |
| 20 | rs13042903 | 4980952 | A | 1.018 | 0.8851 | 1.17 | 0.8063 |
| 20 | rs12479919 | 4980740 | T | 1.017 | 0.8849 | 1.17 | 0.8088 |
| 20 | rs13042159 | 4964124 | G | 1.074 | 0.5763 | 2.003 | 0.8213 |
| 20 | rs6038004 | 4850134 | C | 0.9834 | 0.8466 | 1.142 | 0.8263 |
| 20 | rs6052996 | 4944880 | G | 1.015 | 0.8864 | 1.162 | 0.8312 |
| 20 | rs76476203 | 4909133 | G | 0.9505 | 0.593 | 1.523 | 0.8328 |
| 20 | rs6052993 | 4944838 | G | 1.015 | 0.8863 | 1.162 | 0.8332 |
| 20 | rs6052994 | 4944842 | T | 1.015 | 0.8863 | 1.162 | 0.8332 |
| 20 | rs6052995 | 4944856 | C | 1.015 | 0.8863 | 1.162 | 0.8332 |
| 20 | rs1715378 | 4879837 | A | 0.9792 | 0.8044 | 1.192 | 0.8338 |
| 20 | rs16990309 | 4836440 | T | 0.9773 | 0.7858 | 1.215 | 0.8364 |
| 20 | rs6037991 | 4835868 | A | 1.024 | 0.814 | 1.288 | 0.8399 |
| 20 | rs79530091 | 4915073 | C | 0.9525 | 0.5941 | 1.527 | 0.84 |
| 20 | rs2681107 | 4901872 | C | 1.015 | 0.8631 | 1.194 | 0.8546 |
| 20 | rs2423082 | 4982605 | G | 1.037 | 0.701 | 1.535 | 0.855 |
| 20 | rs1715367 | 4900964 | C | 1.015 | 0.863 | 1.194 | 0.8555 |
| 20 | rs1715364 | 4898896 | T | 1.015 | 0.8619 | 1.196 | 0.8569 |
| 20 | rs75592697 | 4868833 | A | 0.9647 | 0.6525 | 1.426 | 0.8572 |
| 20 | rs6084957 | 4980505 | T | 1.012 | 0.8805 | 1.164 | 0.8636 |
| 20 | rs1715365 | 4899088 | C | 1.014 | 0.8625 | 1.193 | 0.8642 |
| 20 | rs4815759 | 4979886 | A | 1.012 | 0.8802 | 1.163 | 0.8671 |
| 20 | rs1776948 | 4931113 | G | 0.9861 | 0.8345 | 1.165 | 0.8691 |
| 20 | rs11907979 | 4844291 | T | 1.019 | 0.8113 | 1.281 | 0.8695 |
| 20 | rs6052944 | 4843892 | G | 1.019 | 0.8108 | 1.28 | 0.8737 |
| 20 | rs75072655 | 4901895 | A | 0.9769 | 0.7021 | 1.359 | 0.8897 |
| 20 | rs6037998 | 4843887 | T | 0.9848 | 0.7866 | 1.233 | 0.894 |
| 20 | rs1935972 | 4890500 | A | 0.9896 | 0.8423 | 1.163 | 0.8985 |
| 20 | rs6037992 | 4837321 | C | 0.9863 | 0.7938 | 1.225 | 0.9006 |
| 20 | rs3737321 | 4843370 | T | 0.9909 | 0.8521 | 1.152 | 0.905 |
| 20 | rs1715366 | 4899842 | C | 1.009 | 0.8584 | 1.187 | 0.9097 |
| 20 | rs16990312 | 4837063 | G | 0.99 | 0.7977 | 1.229 | 0.9276 |
| 20 | rs6037994 | 4841889 | T | 0.9898 | 0.7896 | 1.241 | 0.9289 |
| 20 | rs6052972 | 4918661 | A | 1.015 | 0.7323 | 1.406 | 0.9298 |
| 20 | rs16990314 | 4837159 | G | 0.9902 | 0.7903 | 1.241 | 0.932 |
| 20 | rs6037993 | 4838525 | C | 0.9903 | 0.7904 | 1.241 | 0.9326 |
| 20 | rs6052942 | 4840591 | T | 1.009 | 0.814 | 1.25 | 0.9378 |
| 20 | rs1776968 | 4893045 | C | 1.005 | 0.8546 | 1.183 | 0.9474 |
| 20 | rs6084932 | 4890548 | C | 0.9953 | 0.8472 | 1.169 | 0.9543 |
| 20 | rs6052940 | 4839890 | A | 1.005 | 0.8102 | 1.246 | 0.9661 |
| 20 | rs6052937 | 4835338 | A | 1.003 | 0.8188 | 1.229 | 0.9749 |
| 20 | rs8125804 | 4885793 | T | 1.004 | 0.7567 | 1.333 | 0.9764 |
| 20 | rs3787455 | 4838270 | A | 1.002 | 0.8614 | 1.164 | 0.9844 |
| 20 | rs6052943 | 4843609 | A | 1.002 | 0.8064 | 1.244 | 0.987 |
| 20 | rs6107541 | 4840270 | C | 0.9995 | 0.806 | 1.239 | 0.9966 |

Note: SNP, Single Nucleotide Polymorphism; Chr, chromosome; BP, Base position; A1, Minor allele; NSCLO, Non-syndromic cleft lip only; OR, odds ratio; L95, Lower-bound of 95% confidence interval; U95, Upper-bound of 95% confidence interval.
