# Supplementary material for: Oxidative stress-mediated apoptosis via the SLC23A2-ascorbic acid interaction contributes to cleft lip development
Source: Front Pediatr. 2025 Oct 2;13:1632778. doi: 10.3389/fped.2025.1632778 (PMC12527864; doi:10.3389/fped.2025.1632778)
Supplement: Supplementary file 4 [file Table4.docx]

**Appendix Table 4** Genotypic association analysis of SNPs at SLC23A2 in NSCLO cases and controls

| CHR | SNP | A1 | A2 | AFF | UNAFF | CHISQ | DF | P |
| --- | --- | --- | --- | --- | --- | --- | --- | --- |
| 20 | rs1105838 | C | A | 142/462/457 | 448/1113/687 | 55.35 | 2 | 9.57E-13 |
| 20 | rs4076098 | G | A | 142/462/457 | 448/1111/689 | 54.68 | 2 | 1.34E-12 |
| 20 | rs13044890 | C | T | 142/461/458 | 446/1111/691 | 54.38 | 2 | 1.55E-12 |
| 20 | rs2203908 | A | G | 143/460/458 | 446/1112/690 | 54.38 | 2 | 1.56E-12 |
| 20 | rs4813726 | G | A | 153/465/443 | 473/1110/665 | 53.26 | 2 | 2.73E-12 |
| 20 | rs2326576 | T | C | 153/465/443 | 473/1107/668 | 52.26 | 2 | 4.48E-12 |
| 20 | rs6053029 | G | C | 112/469/480 | 152/808/1288 | 45.36 | 2 | 1.42E-10 |
| 20 | rs111733047 | T | C | 103/449/509 | 139/790/1319 | 37.1 | 2 | 8.8E-09 |
| 20 | rs6053011 | C | T | 210/547/304 | 591/1142/515 | 22.26 | 2 | 0.0000147 |
| 20 | rs6053012 | C | T | 210/547/304 | 591/1142/515 | 22.26 | 2 | 0.0000147 |
| 20 | rs2203907 | G | C | 307/538/216 | 532/1137/579 | 16.64 | 2 | 0.0002438 |
| 20 | rs2748901 | A | G | 186/520/355 | 310/1081/857 | 11.14 | 2 | 0.003819 |
| 20 | rs1279683 | A | G | 139/445/477 | 322/1051/875 | 10.89 | 2 | 0.004323 |
| 20 | rs6053030 | T | A | 139/445/477 | 320/1052/876 | 10.75 | 2 | 0.004633 |
| 20 | rs55853468 | G | C | 126/431/504 | 305/1009/934 | 10.47 | 2 | 0.005316 |
| 20 | rs113708270 | G | T | 147/448/466 | 344/1049/855 | 10.42 | 2 | 0.005474 |
| 20 | rs2748898 | G | C | 229/533/299 | 408/1095/745 | 10.38 | 2 | 0.005583 |
| 20 | rs6139609 | A | T | 126/430/505 | 305/1005/938 | 10.18 | 2 | 0.006149 |
| 20 | rs6133184 | T | C | 126/430/505 | 305/1005/938 | 10.18 | 2 | 0.006149 |
| 20 | rs6053005 | C | T | 229/534/298 | 412/1093/743 | 10.02 | 2 | 0.006669 |
| 20 | rs2681116 | C | T | 227/536/298 | 410/1095/743 | 9.857 | 2 | 0.007239 |
| 20 | rs6107559 | G | C | 170/510/381 | 288/1047/913 | 9.791 | 2 | 0.00748 |
| 20 | rs13042023 | T | C | 201/547/313 | 364/1109/775 | 9.334 | 2 | 0.0094 |
| 20 | rs6133182 | T | C | 199/546/316 | 370/1096/782 | 8.713 | 2 | 0.01282 |
| 20 | rs6139592 | A | C | 220/536/305 | 403/1094/751 | 8.428 | 2 | 0.01479 |
| 20 | rs1554379 | G | A | 220/536/305 | 403/1095/750 | 8.315 | 2 | 0.01564 |
| 20 | rs6139593 | T | C | 220/536/305 | 404/1094/750 | 8.24 | 2 | 0.01624 |
| 20 | rs6053003 | A | C | 220/536/305 | 404/1094/750 | 8.24 | 2 | 0.01624 |
| 20 | rs13037458 | C | A | 220/536/305 | 405/1095/748 | 7.943 | 2 | 0.01885 |
| 20 | rs7263163 | T | C | 220/536/305 | 406/1094/748 | 7.871 | 2 | 0.01953 |
| 20 | rs77395977 | T | A | 7/225/829 | 34/527/1687 | 6.721 | 2 | 0.03471 |
| 20 | rs1715382 | C | G | 7/225/829 | 34/527/1687 | 6.721 | 2 | 0.03471 |
| 20 | rs1715386 | T | C | 7/215/839 | 32/512/1704 | 6.644 | 2 | 0.03609 |
| 20 | rs1776966 | A | G | 5/214/842 | 25/510/1713 | 6.284 | 2 | 0.04321 |
| 20 | rs4815754 | A | G | 8/219/834 | 33/520/1695 | 5.936 | 2 | 0.0514 |
| 20 | rs2681110 | T | C | 183/538/340 | 342/1095/811 | 5.83 | 2 | 0.0542 |
| 20 | rs2423076 | T | C | 119/466/476 | 213/936/1099 | 5.517 | 2 | 0.06338 |
| 20 | rs1776963 | T | C | 28/319/714 | 92/696/1460 | 4.992 | 2 | 0.0824 |
| 20 | rs1715383 | G | A | 19/268/774 | 57/627/1564 | 4.751 | 2 | 0.09297 |
| 20 | rs1776965 | T | C | 18/262/781 | 53/617/1578 | 4.703 | 2 | 0.09521 |
| 20 | rs2748896 | A | C | 19/281/761 | 62/635/1551 | 4.333 | 2 | 0.1146 |
| 20 | rs1715380 | C | G | 17/271/773 | 55/617/1576 | 4.103 | 2 | 0.1285 |
| 20 | rs138782359 | T | C | 8/128/925 | 10/319/1919 | 3.956 | 2 | 0.1384 |
| 20 | rs2946682 | G | A | 8/129/924 | 10/321/1917 | 3.927 | 2 | 0.1404 |
| 20 | rs78624304 | T | C | 7/108/946 | 14/282/1952 | 3.884 | 2 | 0.1434 |
| 20 | rs1715377 | A | G | 12/244/805 | 39/564/1645 | 3.704 | 2 | 0.1569 |
| 20 | rs939258 | A | T | 8/130/923 | 10/321/1917 | 3.687 | 2 | 0.1583 |
| 20 | rs2748899 | C | T | 152/513/396 | 286/1052/910 | 3.589 | 2 | 0.1662 |
| 20 | rs1715397 | G | A | 6/115/940 | 7/285/1956 | 3.408 | 2 | 0.1819 |
| 20 | rs1776952 | T | A | 6/115/940 | 7/285/1956 | 3.408 | 2 | 0.1819 |
| 20 | rs1715361 | A | G | 6/115/940 | 7/285/1956 | 3.408 | 2 | 0.1819 |
| 20 | rs1776955 | C | T | 100/428/533 | 175/888/1185 | 3.313 | 2 | 0.1908 |
| 20 | rs1715392 | T | C | 6/115/940 | 7/284/1957 | 3.305 | 2 | 0.1915 |
| 20 | rs1519862 | A | C | 6/115/940 | 7/284/1957 | 3.305 | 2 | 0.1915 |
| 20 | rs1519865 | A | G | 6/115/940 | 7/284/1957 | 3.305 | 2 | 0.1915 |
| 20 | rs1715395 | G | A | 6/115/940 | 7/284/1957 | 3.305 | 2 | 0.1915 |
| 20 | rs1776950 | A | G | 6/115/940 | 7/284/1957 | 3.305 | 2 | 0.1915 |
| 20 | rs1971573 | C | T | 123/494/444 | 268/973/1007 | 3.24 | 2 | 0.1979 |
| 20 | rs1776947 | G | A | 6/115/940 | 7/283/1958 | 3.205 | 2 | 0.2014 |
| 20 | rs6038026 | A | G | 151/513/397 | 292/1044/912 | 3.2 | 2 | 0.2019 |
| 20 | rs11907979 | T | C | 7/185/869 | 19/340/1889 | 3.135 | 2 | 0.2086 |
| 20 | rs13045713 | G | C | 46/297/718 | 72/662/1514 | 3.132 | 2 | 0.2089 |
| 20 | rs1519864 | G | A | 6/115/940 | 7/282/1959 | 3.106 | 2 | 0.2116 |
| 20 | rs1343095 | G | C | 7/130/924 | 10/321/1917 | 3.096 | 2 | 0.2127 |
| 20 | rs6052944 | G | A | 7/185/869 | 19/341/1888 | 3.022 | 2 | 0.2207 |
| 20 | rs113257858 | T | C | 6/115/940 | 7/281/1960 | 3.01 | 2 | 0.2221 |
| 20 | rs6139606 | G | A | 100/473/488 | 232/935/1081 | 2.754 | 2 | 0.2523 |
| 20 | rs6037993 | C | T | 8/187/866 | 20/347/1881 | 2.663 | 2 | 0.2641 |
| 20 | rs6037994 | T | A | 8/186/867 | 20/345/1883 | 2.662 | 2 | 0.2642 |
| 20 | rs35560557 | A | G | 15/241/805 | 36/456/1756 | 2.653 | 2 | 0.2654 |
| 20 | rs6116569 | C | T | 76/381/604 | 159/872/1217 | 2.608 | 2 | 0.2715 |
| 20 | rs2681113 | C | T | 154/517/390 | 299/1060/889 | 2.601 | 2 | 0.2724 |
| 20 | rs1401828 | T | G | 99/467/495 | 231/926/1091 | 2.54 | 2 | 0.2808 |
| 20 | rs2748897 | T | C | 149/512/400 | 292/1046/910 | 2.464 | 2 | 0.2917 |
| 20 | rs2748902 | G | A | 154/516/391 | 299/1060/889 | 2.46 | 2 | 0.2923 |
| 20 | rs1891649 | A | G | 68/359/634 | 118/801/1329 | 2.437 | 2 | 0.2956 |
| 20 | rs6139601 | A | C | 106/474/481 | 236/940/1072 | 2.409 | 2 | 0.2998 |
| 20 | rs6139600 | A | G | 106/473/482 | 234/938/1076 | 2.409 | 2 | 0.2999 |
| 20 | rs6133183 | C | A | 106/473/482 | 234/938/1076 | 2.409 | 2 | 0.2999 |
| 20 | rs16990314 | G | A | 9/186/866 | 20/347/1881 | 2.344 | 2 | 0.3098 |
| 20 | rs6053024 | T | G | 106/473/482 | 234/939/1075 | 2.333 | 2 | 0.3115 |
| 20 | rs1776948 | G | A | 34/317/710 | 96/677/1475 | 2.284 | 2 | 0.3192 |
| 20 | rs1776968 | C | A | 40/340/681 | 111/709/1428 | 2.261 | 2 | 0.3228 |
| 20 | rs6038007 | T | C | 72/369/620 | 132/833/1283 | 2.238 | 2 | 0.3266 |
| 20 | rs2681107 | C | T | 41/340/680 | 113/705/1430 | 2.225 | 2 | 0.3287 |
| 20 | rs1715378 | A | G | 12/237/812 | 35/540/1673 | 2.218 | 2 | 0.3298 |
| 20 | rs1715367 | C | T | 41/340/680 | 113/706/1429 | 2.217 | 2 | 0.3301 |
| 20 | rs6053018 | C | G | 106/471/484 | 232/937/1079 | 2.186 | 2 | 0.3352 |
| 20 | rs6053021 | T | C | 106/470/485 | 231/936/1081 | 2.118 | 2 | 0.3468 |
| 20 | rs1969715 | G | A | 106/470/485 | 231/936/1081 | 2.118 | 2 | 0.3468 |
| 20 | rs6139598 | T | C | 106/470/485 | 231/936/1081 | 2.118 | 2 | 0.3468 |
| 20 | rs1543452 | T | C | 106/470/485 | 231/936/1081 | 2.118 | 2 | 0.3468 |
| 20 | rs1519861 | A | T | 106/470/485 | 231/936/1081 | 2.118 | 2 | 0.3468 |
| 20 | rs1715385 | G | A | 74/390/597 | 180/856/1212 | 2.065 | 2 | 0.3561 |
| 20 | rs8115867 | G | A | 68/360/633 | 121/803/1324 | 2.058 | 2 | 0.3573 |
| 20 | rs6116605 | T | C | 106/470/485 | 231/937/1080 | 2.046 | 2 | 0.3595 |
| 20 | rs6116606 | A | G | 106/470/485 | 231/937/1080 | 2.046 | 2 | 0.3595 |
| 20 | rs6053017 | G | C | 106/470/485 | 231/937/1080 | 2.046 | 2 | 0.3595 |
| 20 | rs6053019 | C | T | 106/470/485 | 231/937/1080 | 2.046 | 2 | 0.3595 |
| 20 | rs1715364 | T | C | 39/337/685 | 107/699/1442 | 2.042 | 2 | 0.3603 |
| 20 | rs113321431 | A | G | 106/470/485 | 232/937/1079 | 2.034 | 2 | 0.3616 |
| 20 | rs4987219 | G | C | 71/364/626 | 146/828/1274 | 1.999 | 2 | 0.368 |
| 20 | rs3787457 | T | C | 66/361/634 | 118/806/1324 | 1.997 | 2 | 0.3684 |
| 20 | rs6053016 | C | A | 106/470/485 | 233/938/1077 | 1.956 | 2 | 0.376 |
| 20 | rs6053020 | G | C | 106/470/485 | 233/938/1077 | 1.956 | 2 | 0.376 |
| 20 | rs3787455 | A | G | 72/391/598 | 129/808/1311 | 1.945 | 2 | 0.3781 |
| 20 | rs6084944 | A | T | 154/513/394 | 298/1065/885 | 1.91 | 2 | 0.3849 |
| 20 | rs6052988 | C | T | 150/517/394 | 294/1066/888 | 1.903 | 2 | 0.3862 |
| 20 | rs6053022 | G | A | 106/470/485 | 234/939/1075 | 1.884 | 2 | 0.3898 |
| 20 | rs1715366 | C | G | 42/340/679 | 113/708/1427 | 1.855 | 2 | 0.3955 |
| 20 | rs4813723 | C | T | 77/399/585 | 190/861/1197 | 1.844 | 2 | 0.3977 |
| 20 | rs6053006 | A | G | 142/516/403 | 290/1049/909 | 1.814 | 2 | 0.4037 |
| 20 | rs8125856 | T | C | 8/183/870 | 26/361/1861 | 1.811 | 2 | 0.4043 |
| 20 | rs4815759 | A | G | 100/463/498 | 228/927/1093 | 1.789 | 2 | 0.4088 |
| 20 | rs1776960 | T | C | 74/374/613 | 142/842/1264 | 1.737 | 2 | 0.4197 |
| 20 | rs1715365 | C | T | 42/340/679 | 112/710/1426 | 1.707 | 2 | 0.4259 |
| 20 | rs2298174 | C | G | 64/361/636 | 123/814/1311 | 1.694 | 2 | 0.4288 |
| 20 | rs8122118 | T | C | 67/362/632 | 121/803/1324 | 1.683 | 2 | 0.4312 |
| 20 | rs1715381 | T | A | 72/398/591 | 176/863/1209 | 1.681 | 2 | 0.4314 |
| 20 | rs6139587 | T | A | 148/518/395 | 294/1066/888 | 1.665 | 2 | 0.4349 |
| 20 | rs58061452 | T | G | 143/516/402 | 295/1049/904 | 1.647 | 2 | 0.4389 |
| 20 | rs6052956 | T | C | 69/366/626 | 132/823/1293 | 1.643 | 2 | 0.4397 |
| 20 | rs12480138 | G | C | 144/515/402 | 293/1051/904 | 1.631 | 2 | 0.4424 |
| 20 | rs12481301 | T | A | 144/515/402 | 293/1051/904 | 1.631 | 2 | 0.4424 |
| 20 | rs6084957 | T | C | 100/463/498 | 226/929/1093 | 1.63 | 2 | 0.4426 |
| 20 | rs16990312 | G | A | 12/200/849 | 21/388/1839 | 1.589 | 2 | 0.4517 |
| 20 | rs1776959 | G | A | 74/374/613 | 143/840/1265 | 1.581 | 2 | 0.4536 |
| 20 | rs3914810 | C | T | 68/381/612 | 165/830/1253 | 1.572 | 2 | 0.4558 |
| 20 | rs6038010 | C | T | 71/370/620 | 135/829/1284 | 1.562 | 2 | 0.4578 |
| 20 | rs8123436 | T | C | 65/364/632 | 118/806/1324 | 1.545 | 2 | 0.4618 |
| 20 | rs1923094 | G | A | 109/473/479 | 231/953/1064 | 1.538 | 2 | 0.4634 |
| 20 | rs12479919 | T | C | 101/462/498 | 226/928/1094 | 1.534 | 2 | 0.4644 |
| 20 | rs6139591 | A | G | 143/516/402 | 292/1054/902 | 1.509 | 2 | 0.4703 |
| 20 | rs6053002 | C | T | 143/516/402 | 292/1054/902 | 1.509 | 2 | 0.4703 |
| 20 | rs1879177 | T | C | 147/516/398 | 293/1063/892 | 1.508 | 2 | 0.4704 |
| 20 | rs6052943 | A | G | 11/198/852 | 21/382/1845 | 1.501 | 2 | 0.4721 |
| 20 | rs6038025 | T | C | 146/517/398 | 292/1064/892 | 1.487 | 2 | 0.4754 |
| 20 | rs1715384 | T | A | 74/398/589 | 176/869/1203 | 1.476 | 2 | 0.4782 |
| 20 | rs13042903 | A | G | 101/462/498 | 225/929/1094 | 1.467 | 2 | 0.4803 |
| 20 | rs6052990 | C | T | 147/516/398 | 293/1064/891 | 1.456 | 2 | 0.4829 |
| 20 | rs6038021 | A | G | 147/516/398 | 293/1064/891 | 1.456 | 2 | 0.4829 |
| 20 | rs6052991 | G | A | 147/516/398 | 293/1064/891 | 1.456 | 2 | 0.4829 |
| 20 | rs6052992 | C | T | 147/516/398 | 293/1064/891 | 1.456 | 2 | 0.4829 |
| 20 | rs6139588 | A | C | 147/516/398 | 293/1064/891 | 1.456 | 2 | 0.4829 |
| 20 | rs6052998 | A | G | 147/516/398 | 293/1064/891 | 1.456 | 2 | 0.4829 |
| 20 | rs1879176 | C | T | 147/516/398 | 293/1064/891 | 1.456 | 2 | 0.4829 |
| 20 | rs6052940 | A | T | 11/201/849 | 22/388/1838 | 1.443 | 2 | 0.486 |
| 20 | rs6107541 | C | T | 11/201/849 | 22/388/1838 | 1.443 | 2 | 0.486 |
| 20 | rs6052942 | T | G | 12/201/848 | 23/389/1836 | 1.438 | 2 | 0.4872 |
| 20 | rs6038020 | T | C | 146/517/398 | 293/1064/891 | 1.416 | 2 | 0.4927 |
| 20 | rs6038022 | T | C | 146/517/398 | 293/1064/891 | 1.416 | 2 | 0.4927 |
| 20 | rs6084932 | C | T | 44/336/681 | 114/709/1425 | 1.365 | 2 | 0.5054 |
| 20 | rs1715372 | G | C | 22/267/772 | 61/548/1639 | 1.361 | 2 | 0.5064 |
| 20 | rs6038023 | A | T | 147/515/399 | 293/1064/891 | 1.347 | 2 | 0.5099 |
| 20 | rs1519866 | G | C | 146/515/400 | 291/1064/893 | 1.339 | 2 | 0.5119 |
| 20 | rs6052996 | G | A | 146/518/397 | 299/1061/888 | 1.318 | 2 | 0.5173 |
| 20 | rs3787458 | G | T | 68/364/629 | 126/804/1318 | 1.281 | 2 | 0.527 |
| 20 | rs1935972 | A | C | 44/336/681 | 113/712/1423 | 1.264 | 2 | 0.5315 |
| 20 | rs6052993 | G | C | 146/518/397 | 299/1062/887 | 1.263 | 2 | 0.5318 |
| 20 | rs6052994 | T | C | 146/518/397 | 299/1062/887 | 1.263 | 2 | 0.5318 |
| 20 | rs6052995 | C | G | 146/518/397 | 299/1062/887 | 1.263 | 2 | 0.5318 |
| 20 | rs3787459 | A | C | 63/362/636 | 117/801/1330 | 1.255 | 2 | 0.5338 |
| 20 | rs6037992 | C | G | 11/198/852 | 23/384/1841 | 1.249 | 2 | 0.5355 |
| 20 | rs6037998 | T | C | 9/185/867 | 23/360/1865 | 1.249 | 2 | 0.5356 |
| 20 | rs6038002 | C | T | 72/385/604 | 133/803/1312 | 1.201 | 2 | 0.5486 |
| 20 | rs16990309 | T | C | 9/195/857 | 21/379/1848 | 1.199 | 2 | 0.549 |
| 20 | rs6037991 | A | G | 8/178/875 | 13/350/1885 | 1.174 | 2 | 0.5559 |
| 20 | rs3737321 | T | C | 70/387/604 | 130/809/1309 | 1.05 | 2 | 0.5915 |
| 20 | rs1110277 | G | A | 72/379/610 | 133/816/1299 | 0.9573 | 2 | 0.6196 |
| 20 | rs6052937 | A | C | 12/225/824 | 27/446/1775 | 0.8481 | 2 | 0.6544 |
| 20 | rs1715379 | G | A | 77/410/574 | 176/888/1184 | 0.7217 | 2 | 0.6971 |
| 20 | rs1776964 | G | A | 76/411/574 | 176/881/1191 | 0.6273 | 2 | 0.7308 |
| 20 | rs12481275 | A | T | 71/379/611 | 135/811/1302 | 0.5846 | 2 | 0.7465 |
| 20 | rs12329577 | G | A | 28/264/769 | 51/571/1626 | 0.4929 | 2 | 0.7816 |
| 20 | rs6038004 | C | A | 70/391/600 | 135/826/1287 | 0.4724 | 2 | 0.7896 |
| 20 | rs1715373 | G | A | 81/391/589 | 160/850/1238 | 0.4668 | 2 | 0.7918 |
| 20 | rs1629176 | A | G | 75/381/605 | 147/826/1275 | 0.4507 | 2 | 0.7982 |
| 20 | rs1715374 | G | A | 75/382/604 | 149/826/1273 | 0.3263 | 2 | 0.8495 |
| 20 | rs1715376 | C | T | 76/385/600 | 152/832/1264 | 0.2847 | 2 | 0.8673 |
| 20 | rs1131382 | C | T | 253/531/277 | 546/1104/598 | 0.2531 | 2 | 0.8811 |
| 20 | rs1776956 | G | A | 75/386/600 | 149/826/1273 | 0.2335 | 2 | 0.8898 |
| 20 | rs1715375 | G | A | 75/386/600 | 149/826/1273 | 0.2335 | 2 | 0.8898 |
| 20 | rs1776958 | G | C | 80/394/587 | 163/850/1235 | 0.1895 | 2 | 0.9096 |
| 20 | rs1776957 | T | C | 75/387/599 | 150/826/1272 | 0.1827 | 2 | 0.9127 |
| 20 | rs79137959 | C | G | 4/115/942 | 12/275/1961 | NA | NA | NA |
| 20 | rs146487465 | C | A | 1/104/956 | 3/188/2057 | NA | NA | NA |
| 20 | rs3787456 | A | G | 2/68/991 | 5/205/2038 | NA | NA | NA |
| 20 | rs79181767 | C | T | 2/39/1020 | 1/124/2123 | NA | NA | NA |
| 20 | rs75592697 | A | C | 1/59/1001 | 0/133/2115 | NA | NA | NA |
| 20 | rs1776961 | C | A | 1/20/1040 | 0/53/2195 | NA | NA | NA |
| 20 | rs6052961 | T | C | 3/138/920 | 15/272/1961 | NA | NA | NA |
| 20 | rs6052962 | A | G | 0/39/1022 | 2/98/2148 | NA | NA | NA |
| 20 | rs8125804 | T | C | 3/117/941 | 5/226/2017 | NA | NA | NA |
| 20 | rs74333140 | C | T | 0/38/1023 | 2/96/2150 | NA | NA | NA |
| 20 | rs1715387 | T | G | 0/39/1022 | 2/91/2155 | NA | NA | NA |
| 20 | rs77881678 | T | C | 0/38/1023 | 2/89/2157 | NA | NA | NA |
| 20 | rs1776967 | T | C | 0/39/1022 | 2/93/2153 | NA | NA | NA |
| 20 | rs1614554 | C | T | 0/39/1022 | 2/92/2154 | NA | NA | NA |
| 20 | rs1776970 | C | G | 0/39/1022 | 2/92/2154 | NA | NA | NA |
| 20 | rs1715362 | T | C | 0/39/1022 | 2/92/2154 | NA | NA | NA |
| 20 | rs75072655 | A | G | 1/86/974 | 1/125/2122 | NA | NA | NA |
| 20 | rs3787469 | C | T | 3/96/962 | 3/233/2012 | NA | NA | NA |
| 20 | rs1715368 | C | T | 0/40/1021 | 2/93/2153 | NA | NA | NA |
| 20 | rs74912238 | A | G | 0/39/1022 | 2/93/2153 | NA | NA | NA |
| 20 | rs73897144 | T | A | 0/39/1022 | 2/93/2153 | NA | NA | NA |
| 20 | rs73894175 | G | A | 3/97/961 | 3/235/2010 | NA | NA | NA |
| 20 | rs117968930 | G | C | 3/96/962 | 3/235/2010 | NA | NA | NA |
| 20 | rs1776971 | C | T | 0/43/1018 | 2/95/2151 | NA | NA | NA |
| 20 | rs80244584 | G | A | 3/97/961 | 3/237/2008 | NA | NA | NA |
| 20 | rs1776972 | C | T | 0/43/1018 | 2/95/2151 | NA | NA | NA |
| 20 | rs76415638 | T | C | 3/96/962 | 3/235/2010 | NA | NA | NA |
| 20 | rs1776973 | C | T | 0/43/1018 | 2/95/2151 | NA | NA | NA |
| 20 | rs939257 | C | T | 0/43/1018 | 2/96/2150 | NA | NA | NA |
| 20 | rs939259 | A | C | 0/41/1020 | 2/93/2153 | NA | NA | NA |
| 20 | rs1715370 | T | C | 0/41/1020 | 2/93/2153 | NA | NA | NA |
| 20 | rs76476203 | G | C | 0/40/1021 | 2/91/2155 | NA | NA | NA |
| 20 | rs1776977 | T | C | 0/41/1020 | 2/94/2152 | NA | NA | NA |
| 20 | rs116915305 | A | G | 3/97/961 | 3/235/2010 | NA | NA | NA |
| 20 | rs56671692 | A | G | 3/97/961 | 3/235/2010 | NA | NA | NA |
| 20 | rs59971708 | A | G | 3/97/961 | 3/235/2010 | NA | NA | NA |
| 20 | rs1776978 | A | G | 0/41/1020 | 2/94/2152 | NA | NA | NA |
| 20 | rs73893863 | A | C | 3/97/961 | 4/236/2008 | NA | NA | NA |
| 20 | rs1628664 | A | G | 0/41/1020 | 2/93/2153 | NA | NA | NA |
| 20 | rs3761240 | A | T | 3/97/961 | 3/235/2010 | NA | NA | NA |
| 20 | rs79530091 | C | T | 0/40/1021 | 2/90/2156 | NA | NA | NA |
| 20 | rs55793575 | A | G | 3/97/961 | 3/235/2010 | NA | NA | NA |
| 20 | rs189470744 | T | C | 3/96/962 | 3/235/2010 | NA | NA | NA |
| 20 | rs181793493 | A | G | 0/42/1019 | 2/93/2153 | NA | NA | NA |
| 20 | rs1715360 | C | T | 0/42/1019 | 2/94/2152 | NA | NA | NA |
| 20 | rs6052972 | A | G | 1/91/969 | 5/170/2073 | NA | NA | NA |
| 20 | rs13037855 | T | C | 1/46/1014 | 1/123/2124 | NA | NA | NA |
| 20 | rs111806610 | A | C | 0/19/1042 | 1/67/2180 | NA | NA | NA |
| 20 | rs12625783 | C | T | 0/31/1030 | 0/74/2174 | NA | NA | NA |
| 20 | rs7260796 | C | T | 0/19/1042 | 1/68/2179 | NA | NA | NA |
| 20 | rs57215863 | T | C | 0/19/1042 | 1/68/2179 | NA | NA | NA |
| 20 | rs16990455 | T | G | 0/19/1042 | 1/68/2179 | NA | NA | NA |
| 20 | rs79494287 | A | G | 0/17/1044 | 1/65/2182 | NA | NA | NA |
| 20 | rs113678532 | A | G | 0/18/1043 | 1/69/2178 | NA | NA | NA |
| 20 | rs2681109 | A | G | 2/77/982 | 1/188/2059 | NA | NA | NA |
| 20 | rs75511237 | C | T | 0/17/1044 | 1/65/2182 | NA | NA | NA |
| 20 | rs1776953 | T | C | 0/39/1022 | 0/127/2121 | NA | NA | NA |
| 20 | rs190393511 | C | T | 0/23/1038 | 0/91/2157 | NA | NA | NA |
| 20 | rs35731094 | A | C | 1/85/975 | 5/216/2027 | NA | NA | NA |
| 20 | rs114776261 | T | C | 1/85/975 | 5/216/2027 | NA | NA | NA |
| 20 | rs187777103 | T | G | 1/85/975 | 5/216/2027 | NA | NA | NA |
| 20 | rs201341095 | C | T | 1/85/975 | 5/216/2027 | NA | NA | NA |
| 20 | rs115233603 | T | G | 1/85/975 | 5/216/2027 | NA | NA | NA |
| 20 | rs76480995 | T | C | 1/85/975 | 5/216/2027 | NA | NA | NA |
| 20 | rs79774064 | C | T | 1/85/975 | 5/215/2028 | NA | NA | NA |
| 20 | rs192759748 | T | G | 1/85/975 | 5/215/2028 | NA | NA | NA |
| 20 | rs146463412 | C | T | 1/85/975 | 5/215/2028 | NA | NA | NA |
| 20 | rs577998756 | T | C | 1/84/976 | 5/214/2029 | NA | NA | NA |
| 20 | rs545078767 | C | T | 1/84/976 | 5/214/2029 | NA | NA | NA |
| 20 | rs553897675 | T | C | 1/84/976 | 5/212/2031 | NA | NA | NA |
| 20 | rs572298297 | A | G | 1/84/976 | 5/212/2031 | NA | NA | NA |
| 20 | rs542764191 | T | C | 1/85/975 | 5/215/2028 | NA | NA | NA |
| 20 | rs192725418 | A | G | 1/85/975 | 5/212/2031 | NA | NA | NA |
| 20 | rs531873467 | T | C | 1/85/975 | 5/212/2031 | NA | NA | NA |
| 20 | rs544130388 | G | A | 1/85/975 | 5/212/2031 | NA | NA | NA |
| 20 | rs564704117 | T | C | 1/85/975 | 5/215/2028 | NA | NA | NA |
| 20 | rs532179315 | G | A | 1/85/975 | 5/212/2031 | NA | NA | NA |
| 20 | rs200905767 | G | C | 1/85/975 | 5/212/2031 | NA | NA | NA |
| 20 | rs565742219 | C | T | 1/85/975 | 5/212/2031 | NA | NA | NA |
| 20 | rs78064114 | C | T | 1/85/975 | 5/214/2029 | NA | NA | NA |
| 20 | rs75895094 | T | C | 1/85/975 | 5/217/2026 | NA | NA | NA |
| 20 | rs35653337 | T | C | 1/85/975 | 5/215/2028 | NA | NA | NA |
| 20 | rs79377543 | G | C | 1/85/975 | 5/215/2028 | NA | NA | NA |
| 20 | rs75333496 | C | A | 1/85/975 | 5/215/2028 | NA | NA | NA |
| 20 | rs148303091 | A | T | 1/85/975 | 5/213/2030 | NA | NA | NA |
| 20 | rs115912951 | G | C | 1/85/975 | 5/213/2030 | NA | NA | NA |
| 20 | rs138430286 | T | C | 1/85/975 | 5/213/2030 | NA | NA | NA |
| 20 | rs12625071 | C | A | 1/85/975 | 5/213/2030 | NA | NA | NA |
| 20 | rs144015304 | C | G | 1/85/975 | 5/213/2030 | NA | NA | NA |
| 20 | rs115107415 | A | G | 1/85/975 | 5/213/2030 | NA | NA | NA |
| 20 | rs76434940 | T | C | 1/85/975 | 5/214/2029 | NA | NA | NA |
| 20 | rs79766484 | C | G | 1/85/975 | 5/214/2029 | NA | NA | NA |
| 20 | rs79962391 | T | C | 1/85/975 | 5/214/2029 | NA | NA | NA |
| 20 | rs78629244 | A | G | 2/85/974 | 4/215/2029 | NA | NA | NA |
| 20 | rs76835352 | A | C | 2/85/974 | 4/215/2029 | NA | NA | NA |
| 20 | rs12626128 | T | C | 2/85/974 | 4/215/2029 | NA | NA | NA |
| 20 | rs12626067 | C | G | 2/85/974 | 4/215/2029 | NA | NA | NA |
| 20 | rs12625464 | C | T | 2/85/974 | 4/215/2029 | NA | NA | NA |
| 20 | rs12626153 | T | C | 2/85/974 | 4/215/2029 | NA | NA | NA |
| 20 | rs79054518 | C | G | 2/85/974 | 4/215/2029 | NA | NA | NA |
| 20 | rs76164996 | C | T | 2/85/974 | 4/215/2029 | NA | NA | NA |
| 20 | rs74654281 | G | A | 2/85/974 | 4/215/2029 | NA | NA | NA |
| 20 | rs117554664 | G | A | 2/85/974 | 4/215/2029 | NA | NA | NA |
| 20 | rs36107804 | A | C | 2/50/1009 | 2/114/2132 | NA | NA | NA |
| 20 | rs142637347 | A | G | 2/85/974 | 4/215/2029 | NA | NA | NA |
| 20 | rs150973227 | A | G | 2/85/974 | 4/215/2029 | NA | NA | NA |
| 20 | rs72550899 | C | G | 2/85/974 | 4/215/2029 | NA | NA | NA |
| 20 | rs6053010 | C | T | 0/28/1033 | 1/103/2144 | NA | NA | NA |
| 20 | rs13042159 | G | C | 0/21/1040 | 1/67/2180 | NA | NA | NA |
| 20 | rs6053013 | T | C | 0/36/1025 | 1/47/2200 | NA | NA | NA |
| 20 | rs2681118 | G | T | 0/38/1023 | 1/48/2199 | NA | NA | NA |
| 20 | rs2064842 | C | T | 0/38/1023 | 0/50/2198 | NA | NA | NA |
| 20 | rs58235874 | A | C | 0/38/1023 | 0/50/2198 | NA | NA | NA |
| 20 | rs62200399 | A | G | 0/16/1045 | 4/173/2071 | NA | NA | NA |
| 20 | rs1279682 | T | C | 0/39/1022 | 1/60/2187 | NA | NA | NA |
| 20 | rs2423082 | G | T | 1/57/1003 | 1/118/2129 | NA | NA | NA |
| 20 | rs2423083 | G | A | 1/38/1022 | 0/70/2178 | NA | NA | NA |
| 20 | rs2254964 | G | C | 1/38/1022 | 0/70/2178 | NA | NA | NA |
| 20 | rs2423084 | C | T | 1/38/1022 | 0/70/2178 | NA | NA | NA |
| 20 | rs2423085 | T | G | 0/39/1022 | 0/67/2181 | NA | NA | NA |
| 20 | rs2423086 | G | C | 0/39/1022 | 0/67/2181 | NA | NA | NA |
| 20 | rs6038038 | T | C | 0/38/1023 | 0/62/2186 | NA | NA | NA |

Note: A1, minor allele; A2, major allele; AFF, frequency of genotype in cases; BCL, bilateral cleft lip; CHISQ, chi-square (1df); NSCLO, non-syndromic cleft lip only; p, p-value for this test; SNP, single nucleotide polymorphism; UNAFF, frequency of genotype in controls.
