# Supplementary material for: Oxidative stress-mediated apoptosis via the SLC23A2-ascorbic acid interaction contributes to cleft lip development
Source: Front Pediatr. 2025 Oct 2;13:1632778. doi: 10.3389/fped.2025.1632778 (PMC12527864; doi:10.3389/fped.2025.1632778)
Supplement: Supplementary file 5 [file Table5.docx]

**Appendix Table 5** Association results of SNPs at genes influenced by *SLC23A2*

|  | CHR | GENE | SNP | BP | A1 | *P*_HWE_ | MAF | OR (95%CI) | P |
| --- | --- | --- | --- | --- | --- | --- | --- | --- | --- |
| NSOC | 2 | *IGFBP2* | rs9341191 | 217521648 | T | 0.65 | 0.09 | 0.35（0.28，0.44） | 1.52E-18 |
|  | 17 | *ITGB4* | rs820392 | 73749581 | T | 0.23 | 0.34 | 0.67（0.6，0.75） | 8.10E-13 |
|  | 17 | *ITGB4* | rs820390 | 73747796 | A | 0.57 | 0.35 | 0.7（0.63，0.77） | 1.47E-11 |
|  | 19 | *TLE2* | rs56131688 | 3017613 | T | 0.94 | 0.21 | 0.73（0.65，0.83） | 7.76E-07 |
|  | 17 | *ITGB4* | rs820389 | 73746427 | A | 0.29 | 0.21 | 0.74（0.65，0.84） | 1.51E-06 |
|  | 17 | *ITGB4* | rs820387 | 73746146 | T | 0.19 | 0.22 | 0.74（0.66，0.84） | 1.66E-06 |
|  | 17 | *ITGB4* | rs866581 | 73745899 | T | 0.16 | 0.33 | 0.81（0.73，0.9） | 5.78E-05 |
| NSCL/P | 17 | *ITGB4* | rs820392 | 73749581 | T | 0.23 | 0.34 | 0.64（0.57，0.72） | 5.88E-13 |
|  | 17 | *ITGB4* | rs820390 | 73747796 | A | 0.57 | 0.35 | 0.66（0.59，0.75） | 1.06E-11 |
|  | 2 | *IGFBP2* | rs9341191 | 217521648 | T | 0.65 | 0.09 | 0.46（0.36，0.58） | 1.80E-10 |
|  | 19 | *TLE2* | rs56131688 | 3017613 | T | 0.94 | 0.21 | 0.69（0.6，0.8） | 2.18E-07 |
|  | 17 | *ITGB4* | rs820387 | 73746146 | T | 0.19 | 0.22 | 0.72（0.62，0.82） | 2.18E-06 |
|  | 17 | *ITGB4* | rs820389 | 73746427 | A | 0.29 | 0.21 | 0.72（0.62，0.82） | 3.05E-06 |
|  | 17 | *ITGB4* | rs820388 | 73746370 | T | 0.5 | 0.45 | 0.78（0.7，0.87） | 9.32E-06 |
|  | 17 | *ITGB4* | rs866581 | 73745899 | T | 0.16 | 0.33 | 0.78（0.69，0.87） | 1.72E-05 |
|  | 9 | *NRARP* | rs34679617 | 140195142 | TG | 0.56 | 0.34 | 0.78（0.7，0.88） | 2.86E-05 |
|  | 19 | *TLE2* | rs4061734 | 3000822 | CT | 0.56 | 0.34 | 0.78（0.69，0.88） | 4.15E-05 |
|  | 19 | *TLE2* | rs3760961 | 3013374 | G | 0.41 | 0.42 | 0.81（0.73，0.9） | 9.34E-05 |
| NSCLP | 19 | *TLE2* | rs4061734 | 3000822 | CT | 0.83 | 0.31 | 0.48（0.4，0.58） | 7.96E-15 |
|  | 17 | *ITGB4* | rs820392 | 73749581 | T | 0.23 | 0.34 | 0.49（0.4，0.59） | 3.56E-14 |
|  | 9 | *NRARP* | rs34679617 | 140195142 | TG | 0.56 | 0.34 | 0.51（0.43，0.61） | 6.63E-14 |
|  | 17 | *ITGB4* | rs820390 | 73747796 | A | 0.57 | 0.35 | 0.52（0.43，0.62） | 8.96E-13 |
|  | 19 | *TLE2* | rs56131688 | 3017613 | T | 0.94 | 0.21 | 0.49（0.39，0.61） | 1.68E-10 |
|  | 19 | *TLE2* | rs3760961 | 3013374 | G | 0.41 | 0.42 | 0.62（0.53，0.72） | 1.51E-09 |
|  | 19 | *TLE2* | rs62125445 | 3001390 | C | 0.09 | 0.38 | 0.63（0.54，0.74） | 1.36E-08 |
|  | 19 | *TLE2* | rs10401433 | 3040377 | T | 0.47 | 0.3 | 0.6（0.5，0.72） | 1.42E-08 |
|  | 19 | *TLE2* | rs12611189 | 3042515 | C | 0.78 | 0.31 | 0.6（0.5，0.72） | 1.68E-08 |
|  | 19 | *TLE2* | rs10424408 | 3038440 | A | 0.54 | 0.3 | 0.6（0.51，0.72） | 2.33E-08 |
|  | 19 | *TLE2* | rs10407820 | 3040352 | G | 0.43 | 0.3 | 0.61（0.51，0.72） | 2.44E-08 |
|  | 19 | *TLE2* | rs11084993 | 3042734 | C | 0.96 | 0.31 | 0.61（0.51，0.73） | 2.60E-08 |
|  | 17 | *ITGB4* | rs866581 | 73745899 | T | 0.16 | 0.33 | 0.61（0.51，0.73） | 2.78E-08 |
|  | 19 | *TLE2* | rs28632147 | 3041093 | T | 0.58 | 0.31 | 0.61（0.51，0.72） | 2.82E-08 |
|  | 17 | *ITGB4* | rs820388 | 73746370 | T | 0.5 | 0.45 | 0.64（0.55，0.75） | 3.06E-08 |
|  | 17 | *ITGB4* | rs820387 | 73746146 | T | 0.19 | 0.22 | 0.54（0.43，0.67） | 3.27E-08 |
|  | 19 | *TLE2* | rs4807389 | 3038374 | C | 0.54 | 0.3 | 0.61（0.51，0.73） | 3.65E-08 |
|  | 17 | *ITGB4* | rs820389 | 73746427 | A | 0.29 | 0.21 | 0.54（0.43，0.67） | 5.24E-08 |
|  | 19 | *TLE2* | rs199521660 | 2999724 | CA | 0.51 | 0.24 | 0.58（0.48，0.71） | 9.78E-08 |
|  | 19 | *TLE2* | rs34462423 | 3036740 | TCA | 0.61 | 0.3 | 0.62（0.52，0.74） | 1.17E-07 |
|  | 19 | *TLE2* | rs66751326 | 3031286 | C | 0.27 | 0.29 | 0.62（0.52，0.75） | 2.49E-07 |
|  | 5 | *FLT4* | rs115620062 | 180051710 | C | 1 | 0.25 | 0.6（0.5，0.73） | 2.77E-07 |
|  | 5 | *FLT4* | rs2242211 | 180049432 | A | 0.81 | 0.26 | 0.61（0.51，0.74） | 2.80E-07 |
|  | 19 | *TLE2* | rs4807388 | 3031512 | C | 0.25 | 0.29 | 0.63（0.52，0.75） | 2.89E-07 |
|  | 5 | *FLT4* | rs144453562 | 180052831 | T | 1 | 0.24 | 0.6（0.5，0.73） | 3.04E-07 |
|  | 19 | *TLE2* | rs9304902 | 3030370 | A | 0.53 | 0.3 | 0.63（0.53，0.75） | 3.63E-07 |
|  | 19 | *TLE2* | rs112399902 | 3032102 | C | 0.3 | 0.29 | 0.63（0.53，0.75） | 4.06E-07 |
|  | 5 | *FLT4* | rs146943555 | 180035069 | A | 0.42 | 0.2 | 0.58（0.47，0.72） | 6.76E-07 |
|  | 5 | *FLT4* | rs728986 | 180051118 | T | 0.67 | 0.34 | 0.66（0.56，0.78） | 1.03E-06 |
|  | 5 | *FLT4* | rs74201322 | 180030996 | A | 0.88 | 0.19 | 0.59（0.47，0.73） | 1.42E-06 |
|  | 5 | *FLT4* | rs307835 | 180033605 | T | 0.31 | 0.2 | 0.61（0.49，0.75） | 2.50E-06 |
|  | 5 | *FLT4* | rs2927582 | 180041274 | T | 0.31 | 0.2 | 0.61（0.49，0.75） | 2.99E-06 |
|  | 5 | *FLT4* | rs307834 | 180033738 | G | 0.34 | 0.2 | 0.61（0.5，0.75） | 3.30E-06 |
|  | 5 | *FLT4* | rs366388 | 180041008 | T | 0.31 | 0.2 | 0.61（0.5，0.75） | 3.47E-06 |
|  | 5 | *FLT4* | rs384861 | 180041637 | G | 0.31 | 0.2 | 0.61（0.5，0.75） | 3.52E-06 |
|  | 5 | *FLT4* | rs389528 | 180037928 | G | 0.42 | 0.2 | 0.61（0.5，0.75） | 4.06E-06 |
|  | 5 | *FLT4* | rs451207 | 180037929 | T | 0.42 | 0.2 | 0.61（0.5，0.75） | 4.06E-06 |
|  | 5 | *FLT4* | rs307829 | 180037940 | C | 0.42 | 0.2 | 0.61（0.5，0.75） | 4.06E-06 |
|  | 5 | *FLT4* | rs421662 | 180041001 | G | 0.31 | 0.2 | 0.62（0.5，0.76） | 4.72E-06 |
|  | 5 | *FLT4* | rs307833 | 180035504 | C | 0.34 | 0.2 | 0.62（0.5，0.76） | 5.21E-06 |
|  | 5 | *FLT4* | rs449774 | 180045401 | T | 0.38 | 0.2 | 0.62（0.5，0.76） | 5.52E-06 |
|  | 17 | *ITGB4* | rs1008177 | 73751990 | T | 0.16 | 0.5 | 0.71（0.61，0.82） | 5.86E-06 |
|  | 5 | *FLT4* | rs11249739 | 180040457 | T | 0.41 | 0.19 | 0.61（0.5，0.76） | 6.57E-06 |
|  | 5 | *FLT4* | rs307831 | 180037644 | T | 0.31 | 0.2 | 0.62（0.51，0.77） | 7.32E-06 |
|  | 7 | *LFNG* | rs10261289 | 2554729 | C | 0.66 | 0.2 | 0.63（0.51，0.77） | 7.67E-06 |
|  | 5 | *FLT4* | rs100659 | 180039187 | A | 0.31 | 0.2 | 0.63（0.51，0.77） | 9.71E-06 |
|  | 20 | *COL9A3* | rs2294995 | 61468571 | T | 0.24 | 0.3 | 0.7(0.59，0.82) | 1.91E-05 |
|  | 5 | *FLT4* | rs3797104 | 180051987 | C | 1 | 0.25 | 0.67（0.56，0.81） | 2.53E-05 |
|  | 9 | *LAMC3* | rs3780275 | 133913434 | G | 0.47 | 0.13 | 0.61（0.48，0.77） | 3.86E-05 |
|  | 19 | *TLE2* | rs1688134 | 3044901 | G | 0.49 | 0.22 | 1.41（1.2，1.66） | 4.01E-05 |
|  | 7 | *LFNG* | rs375386359 | 2554677 | T | 0.51 | 0.2 | 0.65（0.53，0.8） | 4.56E-05 |
|  | 5 | *FLT4* | rs564487821 | 180051227 | GT | 0.53 | 0.44 | 0.73（0.63，0.85） | 4.58E-05 |
|  | 5 | *FLT4* | rs3822406 | 180054853 | T | 0.89 | 0.47 | 0.74（0.64，0.86） | 6.36E-05 |
| NSCLO | 2 | *IGFBP2* | rs9341191 | 217521648 | T | 0.65 | 0.09 | 0.2（0.13，0.29） | 5.33E-16 |
|  | 17 | *ITGB4* | rs820392 | 73749581 | T | 0.23 | 0.34 | 0.73（0.63，0.85） | 2.23E-05 |
|  | 17 | *ITGB4* | rs820390 | 73747796 | A | 0.57 | 0.35 | 0.74（0.65，0.86） | 3.32E-05 |
| NSCPO | 2 | *IGFBP2* | rs9341191 | 217521648 | T | 0.65 | 0.09 | 0.05（0.03，0.11） | 1.09E-15 |

Note: SNPs with p<1E-04 are displayed in the table. SNP, Single Nucleotide Polymorphism; Chr, chromosome; BP, Base position; A1, Minor allele; NSCL/P, Non-syndromic cleft lip with or without palate; NSCLP, Non-syndromic cleft lip and palate; NSCLO, Non-syndromic cleft lip only; NSCPO, Non-syndromic cleft palate only; OR, odds ratio; 95%CI, 95% confidence interval.
