# Supplementary material for: Oxidative stress-mediated apoptosis via the SLC23A2-ascorbic acid interaction contributes to cleft lip development
Source: Front Pediatr. 2025 Oct 2;13:1632778. doi: 10.3389/fped.2025.1632778 (PMC12527864; doi:10.3389/fped.2025.1632778)
Supplement: Supplementary file 7 [file Table7.docx]

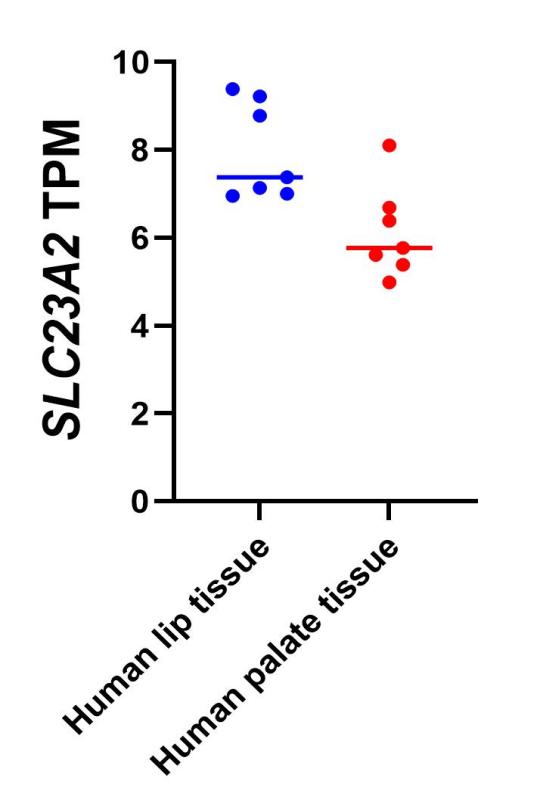


Appendix Fig 1. The expression of SLC23A2 in Human lip and palate tissue.


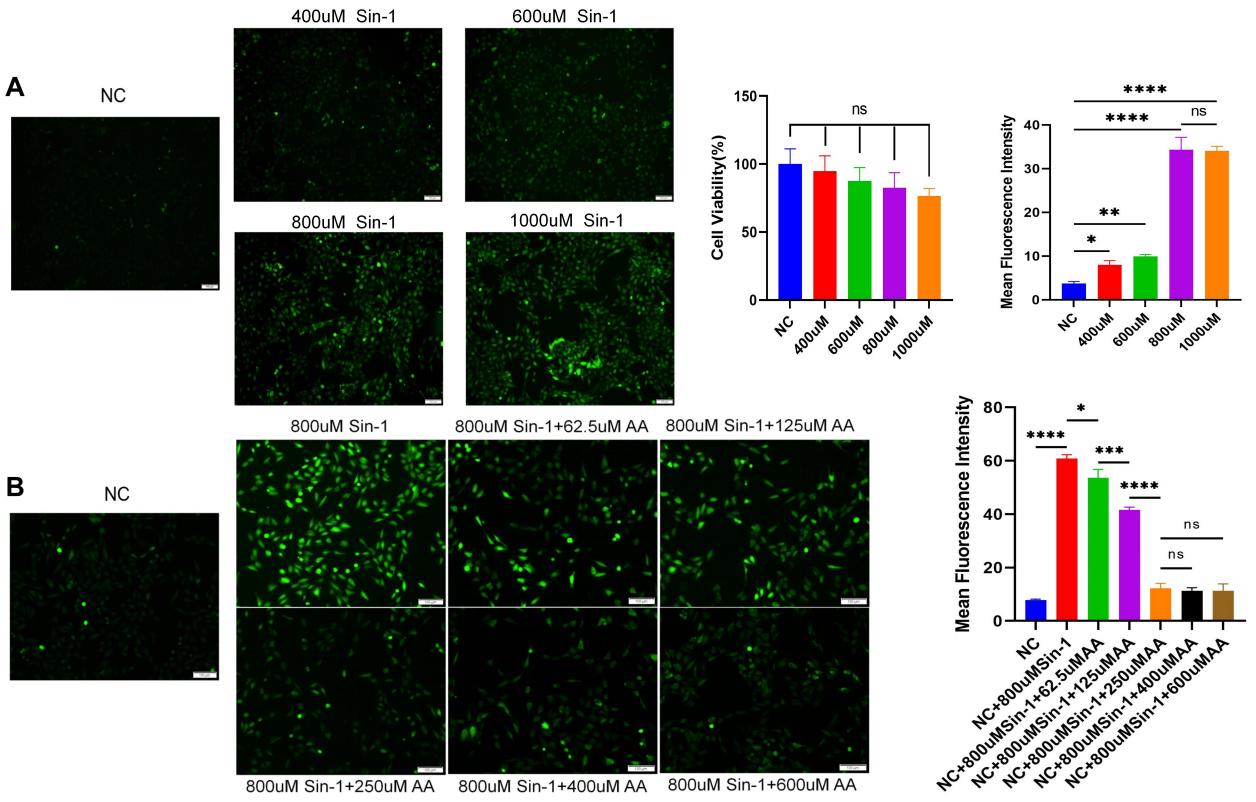


Appendix Fig 2. Detection of ROS in GMSM-K under the action of Sin-1 and AA. A. Detection of cell viability and ROS in GMSM-K under different concentrations of Sin-1. B. Detection of ROS in GMSM-K under different concentrations of AA.
