# Supplementary material for: Oxidative stress-mediated apoptosis via the SLC23A2-ascorbic acid interaction contributes to cleft lip development
Source: Front Pediatr. 2025 Oct 2;13:1632778. doi: 10.3389/fped.2025.1632778 (PMC12527864; doi:10.3389/fped.2025.1632778)
Supplement: Supplementary file 8 [file Datasheet1.docx]

**Primers of small interfering RNA targeting *SLC23A2***

| siRNA/Gene | primers（5’-3’） |
| --- | --- |
| si-SLC23A2 Ⅰ | F:CCUCUCCCGAUUAUTTUAUAA |
|  | R:AUUUAUAAAUCGGGAGAGGTT |
| si-SLC23A2Ⅱ | F:GAGCCAUCCUGUCUUUAGATT |
|  | R:UCUAAAGACAGGAUGGCUCTT |
| si-SLC23A2Ⅲ | F:CCAGCAUCAUCGAGUCUAUTT |
|  | R:AUAGACUCGAUGAUGCUGGTT |
| si-NC | F:UUCUCCGAACGUGUCACGUTT |
|  | R:ACGUGACACGUUCGGAGAATT |
| *SLC23A2* | F:AACACCACAGATGTTTCAGTTG |
|  | R:GGACCGATGTACTTCAGTAGAG |
| *GAPDH* | F:CTTTGGTATCGTGGAAGGACTC |
|  | R:GTAGAGGCAGGGATGATGTTCT |


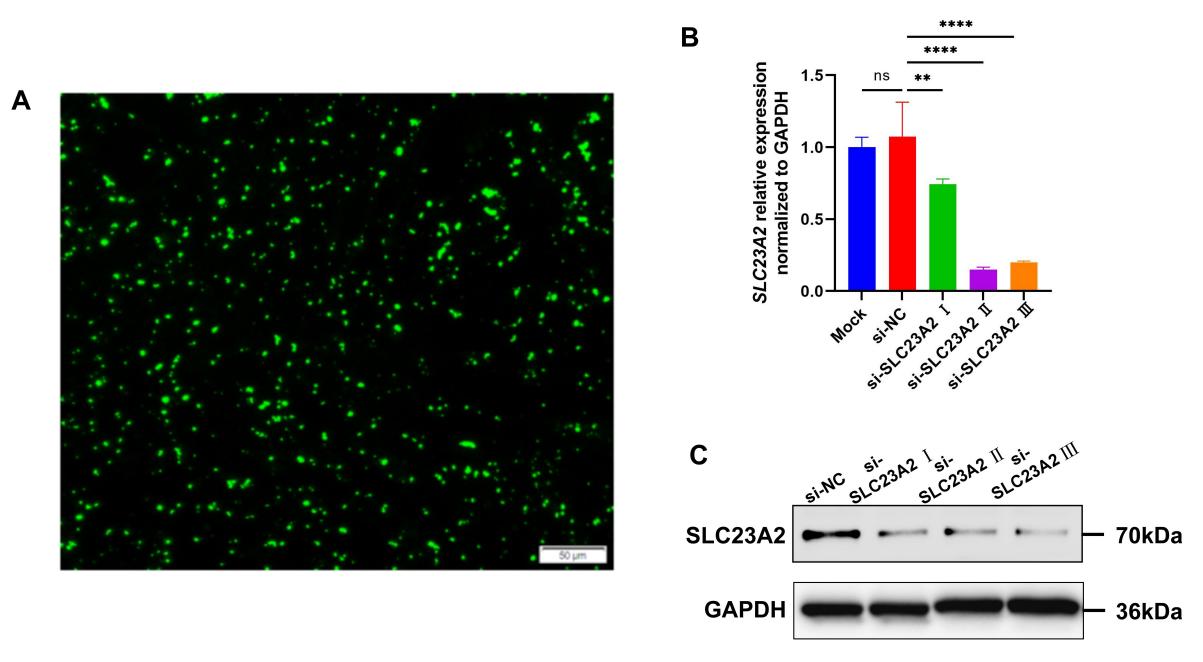


(A) Transfected siRNA into cells (B-C) Transfection efficiency was measured by qPCR and WB,respectively. Error bars represent SD. n=3；ns, P>0.05; *,P < 0.05; **,P < 0.01; ***,P < 0.001; ****,P<0.0001.Scale bar, 50um.

We initially designed three siRNAs. Based on the qPCR and WB data, we chose si-SLC23A2 II. This is the data from our previous experiments.

The image showed the SLC23A2 and its homologs expression data obtained from RNA sequencing. Expression levels of SLC23A1 and SLC23A3 are extremely low in our cell line
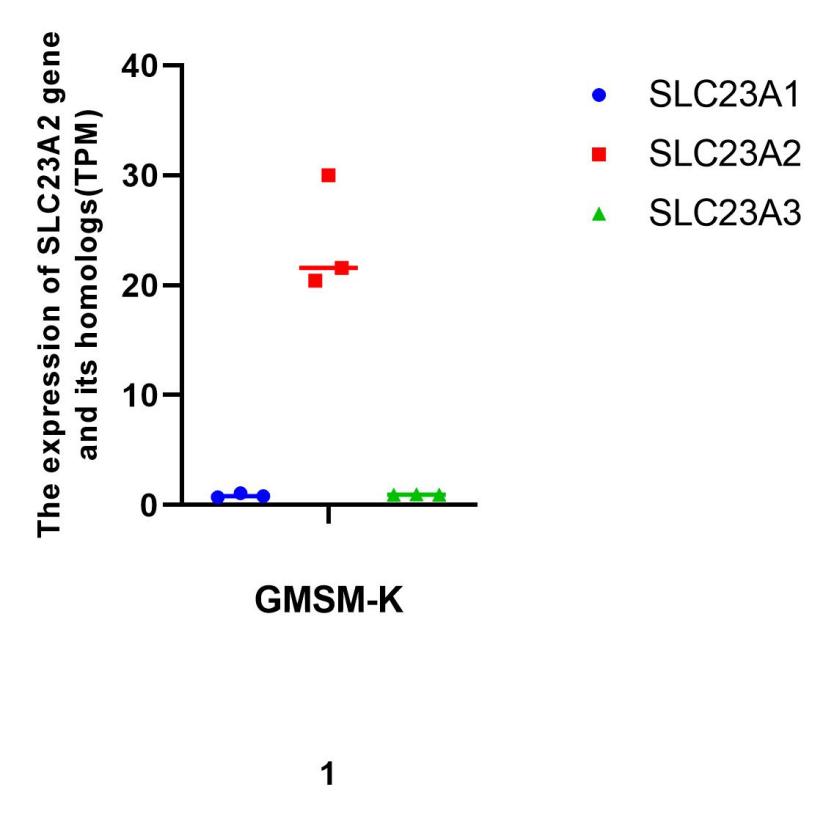
. Therefore, they will not compensate for the knockdown of SLC23A2.
